# Elevated serum chemokine CCL22 levels in first-episode psychosis: associations with symptoms, peripheral immune state and in vivo brain glial cell function

**DOI:** 10.1038/s41398-020-0776-z

**Published:** 2020-03-16

**Authors:** Heikki Laurikainen, Arja Vuorela, Anna Toivonen, Linnea Reinert-Hartwall, Kalevi Trontti, Maija Lindgren, Jaakko Keinänen, Teemu Mäntylä, Janina Paju, Tuula Ilonen, Reetta-Liina Armio, Maija Walta, Jouni Tuisku, Semi Helin, Päivi Marjamäki, Iiris Hovatta, Sebastian Therman, Outi Vaarala, Outi Linnaranta, Tuula Kieseppä, Raimo K. R. Salokangas, Jarno Honkanen, Jarmo Hietala, Jaana Suvisaari

**Affiliations:** 1 Turku PET Centre, University of Turku, Turku, Finland; 2 Department of Psychiatry, University of Turku and Turku University Hospital, Turku, Finland; 3 Clinicum, University of Helsinki, Helsinki, Finland; 4 Research Program for Clinical and Molecular Metabolism, University of Helsinki, Helsinki, Finland; 5 SleepWell Research Program, University of Helsinki, Helsinki, Finland; 6 Department of Psychology and Logopedics, University of Helsinki, Helsinki, Finland; 7 Finnish Institute for Health and Welfare, Mental Health Unit, Helsinki, Finland; 8 Douglas Mental Health University Institute, Montreal, QC Canada; 9 Department of Psychiatry, McGill University, Montreal, QC Canada; 10 Department of Psychiatry, Helsinki University and Helsinki University Hospital, Helsinki, Finland

**Keywords:** Molecular neuroscience, Schizophrenia, Bipolar disorder

## Abstract

Several lines of research support immune system dysregulation in psychotic disorders. However, it remains unclear whether the immunological marker alterations are stable and how they associate with brain glial cell function. This longitudinal study aimed at investigating whether peripheral immune functions are altered in the early phases of psychotic disorders, whether the changes are associated with core symptoms, remission, brain glial cell function, and whether they persist in a one-year follow-up. Two independent cohorts comprising in total of 129 first-episode psychosis (FEP) patients and 130 controls were assessed at baseline and at the one-year follow-up. Serum cyto-/chemokines were measured using a 38-plex Luminex assay. The FEP patients showed a marked increase in chemokine CCL22 levels both at baseline (*p* < 0.0001; Cohen’s *d* = 0.70) and at the 12-month follow-up (*p* = 0.0007) compared to controls. The group difference remained significant (*p* = 0.0019) after accounting for relevant covariates including BMI, smoking, and antipsychotic medication. Elevated serum CCL22 levels were significantly associated with hallucinations (*ρ* = 0.20) and disorganization (*ρ* = 0.23), and with worse verbal performance (*ρ* = −0.23). Brain glial cell activity was indexed with positron emission tomography and the translocator protein radiotracer [^11^C]PBR28 in subgroups of 15 healthy controls and 14 FEP patients with serum CCL22/CCL17 measurements. The distribution volume (*V*_T_) of [^11^C]PBR28 was lower in patients compared to controls (*p* = 0.026; Cohen’s *d* = 0.94) without regionally specific effects, and was inversely associated with serum CCL22 and CCL17 levels (*p* = 0.036). Our results do not support the over-active microglia hypothesis of psychosis, but indicate altered CCR4 immune signaling in early psychosis with behavioral correlates possibly mediated through cross-talk between chemokine networks and dysfunctional or a decreased number of glial cells.

## Introduction

Psychotic disorders are associated with a multitude of immunological changes, including elevations of inflammatory cytokines and chemokines in blood and cerebrospinal fluid^1–3^, as well as changes in central nervous system (CNS) immune cell function^4^. Many changes have been reported in the acute phase of psychosis, but some inflammatory markers remain persistently elevated over the course of illness suggesting a specific link between immunological factors and psychotic disorders^1^. It has been suggested that 30–40% of individuals with schizophrenia or schizoaffective disorder have a inflammatory subtype^5,6^, associating with more severe cognitive difficulties and brain structural abnormalities^5^.

According to the microglial activation hypothesis of schizophrenia, an excessive microglial activation associates with brain morphological and functional changes in patients with schizophrenia^7,8^. The 18-kDa translocator protein (TSPO), a suggested marker for brain microglial cell activation, has been widely studied with positron emission tomography (PET) in psychotic disorders to test the microglial hypothesis of schizophrenia^9–12^. Both increased, unchanged, and decreased brain TSPO measures have been reported in patients with psychosis with different directions of effect seemingly depending on the properties of used radiotracers^11–14^. A recent meta-analysis of in vivo studies using second generation TSPO radiotracers in psychosis patients reports a lower expression of brain TSPO in patients compared to controls^11^, while transcriptomic evidence shows downregulation of microglia related genes in schizophrenia^15^. These observations are not in line with the hypothesis of excessive microglial activation in schizophrenia. Further, the cellular origin of reduced TSPO expression in absence of robust immune stimulation is largely unresolved^16^. Associating TSPO tracer *V*_T_ to auxiliary immune parameters, such as cyto/chemokine networks, have been suggested to facilitate the interpretation of the source and functional association of variation in TSPO *V*_T_^10^.

We have previously studied a panel of 38 cytokines and chemokines in patients with first-episode psychosis (FEP) and healthy controls, and found elevated serum chemokine CCL22 levels associating with white matter volume and diffusion in FEP patients^17^. Here, we used two Finnish cohorts of FEP patients and matched controls to study (a) differences in serum cytokine and chemokine levels in FEP patients and controls, (b) the role of potential confounding factors, namely age, sex, smoking, cannabis use, body mass index (BMI), and antipsychotic medication type, duration, and dose, (c) the persistence of any changes during one-year follow-up, and (d) the relationship between the observed changes and clinical characteristics such as the severity of symptoms and achieving remission in the one-year follow-up. We further tested (e) differences in brain glial cell function in a subsample of recent onset FEP patients and healthy controls using the second generation TSPO radiotracer [^11^C]PBR28 and PET, and (f) importantly, whether changes in serum levels of immune markers associate with brain glial cell function.

## Subjects and methods

### Participants

We recruited two independent geographically distinct cohorts of in- or outpatients with first treatment contact for a psychotic episode (FEP) between December 2009 and November 2017. In the Helsinki Early Psychosis Study (HEPS), patients were recruited from the clinics of the City of Helsinki and the Helsinki University Hospital, and in the Turku Early Psychosis Study (TEPS), from the clinics of the City of Turku and the Hospital district of Southwest Finland. All primary psychotic disorders were included, whereas substance-induced psychoses and psychotic disorders due to a general medical condition were excluded. Controls, matched by age, sex, and place of residence, were recruited through the Population Register Centre. All participants were 18–40 years of age.

In the current study, we included 129 patients (*N* = 82 in the HEPS; *N* = 47 in the TEPS) and 130 controls (*N* = 49 in the HEPS; *N* = 81 in the TEPS) for whom unthawed serum samples were available. A subgroup of first-episode patients (*N* = 14) and healthy controls (HC) (*N* = 15) were included from the TEPS study for a [^11^C]PBR28 PET study. All PET study subjects underwent a structural MRI scan with the Philips 3T Ingenuity PET/MR hybrid scanner to exclude any structural abnormalities.

A detailed description of the inclusion and exclusion criteria used in each sample can be found in the Supplementary Methods. This study was performed in compliance with the 6^th^ revision of the Declaration of Helsinki^18^. The HEPS and TEPS were approved by the Ethics Committees of the Hospital Districts of Helsinki and Uusimaa (diary numbers 257/12/03/03/2009 and 226/13/03/03/2013), and Southwest Finland (diary numbers 64/180/2011 and 65/1801/2013) respectively. All participants gave written informed consent before participation. Capacity to consent was assessed by the patient’s responsible clinician.

### Laboratory analytical methods

Fasting blood samples were collected at 8–10 a.m. Serum samples coagulated at room temperature (max. 2 h) and were then centrifuged, aliquoted and stored at −80 °C. For the PET study, fasting serum was sampled before radiotracer injection at 8–9 a.m. on the day of PET. The HEPS sample included 34 patients and 17 controls from our previous report^17^, but we used unthawed serum samples from these participants and reanalyzed them as a part of the current project.

The serum concentrations of cytokines, chemokines and growth factors (listed in Table 1) were analyzed using the 38-plexed Milliplex MAP Kit (cat. no. HCYTMAG-60K-PX38) according to the manufacturer’s recommendations (Merck-Millipore, Billerica, MA, USA). Additionally, unthawed serum from the PET sample was reanalyzed and complemented with analyzes of CCL17 levels based on the association of elevated CCR4 receptor ligand CCL22 with FEP in the complete sample. CCL22 levels of PET subjects were reanalyzed using the same 38-plexed Milliplex plate to avoid methodological bias, while CCL17 levels were analyzed in duplicate reactions using a 1:2 dilution of the serum samples with the commercial Human CCL17 (TARC) ELISA kit according to the manufacturer’s instructions (Thermo Scientific, Waltham, Massachusetts, USA). Details on the quantification of cyto- and chemokines can be found in the Supplementary Methods.

**Table 1:** Baseline serum cytokine and chemokine levels in patients with first-episode psychosis and controls.

	Patients (*N* = 129) Md (Q1, Q3)	Controls (*N* = 130) Md (Q1, Q3)	*p*-Value (Wilcoxon two-sample test)
CCL2	701.3 (557.3, 877.5)	649.5 (521.7, 800.8)	0.08
CCL3	23.1 (12.0, 35.7)	20.2 (9.4, 35.2)	0.46
CCL4	62.8 (36.0, 130.6)	69.1 (29.0, 131.3)	0.61
CCL7	30.2 (1.9, 190.3)	37.5 (1.9, 253.3)	0.60
CCL11	166.5 (120.5, 219.1)	145.2 (120.0, 197.6)	0.08
CCL22	1277.5 (1014.6, 1625.3)	955.7 (726.8, 1199.0)	**<0.0001**
CX3CL1	48.4 (11.4, 143.1)	61.3 (11.4, 206.5)	0.53
CXCL1	990.1 (750.8, 1257.2)	1022.9 (852.0, 1330.0)	0.13
CXCL10	245.3 (180.6, 297.4)	217.1 (176.1, 279.0)	0.19
EGF	130.0 (83.1, 211.1)	133.7 (85.7, 203.9)	0.90
FGF-2	74.8 (32.3, 134.0)	76.7 (40.3, 134.7)	0.52
FLT-3L	2.7 (2.7, 18.5)	2.7 (2.7, 19.9)	0.63
G-CSF	38.5 (0.9, 73.2)	27.1 (0.9, 87.2)	0.38
GM-CSF	3.8 (3.8, 25.2)	3.8 (3.8, 23.6)	0.95
IFN-α2	8.5 (1.5, 52.8)	10.0 (1.5, 58.0)	0.63
IFN-γ	17.4 (6.5, 56.5)	22.7 (8.3, 68.0)	0.27
IL-1α	24.8 (4.7, 121.2)	23.0 (4.7, 140.3)	0.80
IL-1β	1.0 (0.4, 3.7)	0.4 (0.4, 3.4)	0.25
IL-1RA	13.6 (4.2, 179.8)	22.7 (4.2, 248.0)	0.74
IL-2	4.7 (0.5, 14.7)	1.2 (0.5, 19.5)	0.55
IL-3	0.4 (0.4, 3.9)	0.4 (0.4, 3.6)	0.87
IL-4	2.3 (2.3, 11.7)	2.3 (2.3, 7.3)	0.96
IL-5	1.5 (0.3, 15.2)	1.1 (0.3, 22.5)	0.77
IL-6	11.0 (0.5, 34.8)	12.9 (0.5, 41.9)	0.59
IL-7	3.1 (0.7, 12.4)	2.7 (0.7, 15.0)	0.50
IL-8	29.0 (13.2, 64.8)	28.8 (11.7, 62.1)	0.83
IL-9	3.1 (0.6, 8.9)	0.6 (0.6, 11.0)	0.30
IL-10	5.5 (0.6, 29.2)	0.6 (0.6, 29.2)	0.20
IL12-p40	3.7 (3.7, 117.1)	3.7 (3.7, 105.5)	0.87
IL12-p70	5.8 (0.3, 54.0)	11.0 (0.3, 38.9)	0.71
IL-13	24.6 (0.7; 149.1)	25.1 (0.7, 166.0)	0.85
IL-15	6.0 (0.6, 20.5)	1.6 (0.6, 20.8)	0.41
IL-17	15.1 (4.7, 46.0)	17.3 (7.9, 43.2)	0.31
sCD40L	3150.7 (2186.6, 4228.1)	3107.5 (2298.1, 4035.8)	0.93
TGF-α	7.9 (3.5, 13.6)	7.4 (4.1, 14.7)	0.63
TNF-α	14.9 (11.1, 20.4)	12.0 (10.2, 18.0)	**0.019**
TNF-β	0.8 (0.8, 154.3)	0.8 (0.8, 205.1)	0.55
VEGF	306.4 (165.5, 525.8)	280.1 (128.9, 485.5)	0.40

CCL2 = C-C motif chemokine 2 (MCP-1, monocyte chemoattractant protein 1), CCL3 = macrophage inflammatory protein 1-alpha (MIP-1α); CCL4 = macrophage inflammatory protein 1-beta (MIP-1β); CCL7 = monocyte-chemotactic protein 3 (MCP-3); CCL11 = eotaxin; CCL22 = macrophage derived chemokine; CX3CL1 = chemokine (C-X3-C motif) ligand = fractalcine; CXCL1 = chemokine (C-X-C motif) ligand 1 = GROα; CXCL10 = IP10 (Interferon gamma-induced protein 10)*. Md* median, *Q1* first quartile, *Q3* third quartile, *EGF* epidermal growth factor, *FGF-2* basic fibroblast growth factor, *FLT-3L* Fms-related tyrosine kinase 3 ligand, *G-CSF* granulocyte-colony stimulating factor, *GM-CSF* human granulocyte-macrophage colony–stimulating factor, *IFN* interferon, *IL* interleukin, *IL-1RA* interleukin 1 receptor antagonist, *IL-8* chemokine (C-X-C motif) ligand 8, *IL12-p40* subunit beta of interleukin 12 (common subunit for IL-12 and IL-23; IL12B), *IL12-p70* the active heterodimer of IL-12, *sCD40L* soluble CD-40 ligand, *TGF-α* transforming growth factor alpha, *TNF-α* tumor necrosis factor-alpha, *TNF-β* tumor necrosis factor-beta, *VEGF* vascular endothelial growth factor.

Bold values indicate *p* < 0.05.

### Potential confounding factors

We examined the following variables as potential confounding factors that could explain differences between patients and controls in serum cytokine and chemokine levels: age and sex^19^, BMI^20^, smoking^21^, cannabis use^22^, sample storage time^23^, and antipsychotic medication, analyzing the potential effects of different antipsychotics and the chlorpromazine equivalent dose^24^.

### Clinical measures

Severity of symptoms (past week) was assessed in the HEPS and the PET study using the Brief Psychiatric Rating Scale—Extended (BPRS-E) complemented by 3 domains (alogia, anhedonia-asociality and avolition-apathy) from the Scale for the Assessment of Negative Symptoms (SANS)^25,26^. In the TEPS patients were interviewed with the Positive and Negative Syndrome Scale (PANSS)^27^. Remission status at follow-up was defined using the Andreasen et al. criteria^28^. Social functioning was assessed using the Social and Occupational Functioning Assessment Scale (SOFAS)^29^. Baseline cognitive functioning was assessed in both centers using a harmonized protocol including tests for verbal and visuomotor domains. See Supplementary Methods and Supplementary Tables 1 and 2 for details.

### Positron emission tomography

Immunological stimuli seem to be able to affect both the plasma tracer concentrations and brain uptake of [^11^C]PBR28^30^. Thus distribution volume (*V*_T_) was used as an index of brain glial cell function^30^. Tracer binding to TSPO is dependent on presence of a single nucleotide variation (rs6971) of the TSPO gene^31^. Here DNA was extracted using the Qiagen QIAsymphony DSP DNA mini kit, and TSPO genotyping was performed using a TaqMan Allelic Discrimination assay^32^. This determines homozygote or heterozygote rs6971 carrier status associating with low affinity (LAB), medium affinity (MAB) or high affinity binder (HAB) genotypes^32^. Due to substantial metabolism of [^11^C]PBR28 to radioactive metabolites, and a lack of a suitable reference region in the brain without specific TSPO binding, metabolite corrected arterial plasma input was used for modeling of [^11^C]PBR28 *V*_T_^33^. The *V*_T_ estimates of [^11^C]PBR28 are highly reliable and moderately reproducible when using either region of interest (ROI) two-tissue compartmental modeling, or voxel-wise approaches^34^. Age, sex, BMI, and diurnal changes have been reported as potential sources of bias^34,35^.

The tracer [^11^C]PBR28 was given as an intravenous bolus injection. Emission data were gathered in 3D list-mode first for 70 min with the GE D690 PET/CT scanner (GE Healthcare systems, Chicago, Illinois, USA). Tracer injections were given between 10–11 a.m. in all but one subject^34^. Mean time activity curves were derived from 17 volumes of interest (VOI) of individual T1 weighted MRI segmentations using freesurfer (http://surfer.nmr.mgh.harvard.edu/), and from a composite VOI including all 17 VOIs to assess global gray matter *V*_T_. Regional time-activity curves and metabolite corrected arterial input was used to calculate [^11^C]PBR28 *V*_T_ using 2-tissue compartment modeling with blood volume fixed at 5%^33,34^. Modeling was done with PMOD 3.4 software (PMOD Technologies, Zurich, Switzerland) using Poisson weighting of residuals.

### Statistical analyses of serum concentrations of cytokines, chemokines and growth factors

The HEPS, TEPS and PET study samples were combined into inclusive FEP patient and control groups. We compared the levels of cytokines and chemokines between all FEP patients and controls, and analyzed the stability of these levels between baseline and one-year follow-up using the nonparametric Wilcoxon signed-rank test with *t* approximation of *p*-values. Comparisons in sociodemographic and clinical variables were done with the same test for continuous or ordinal variables, and with Pearson’s *χ*^2^ test for dichotomous variables. Correlations of cytokines and chemokines with clinical variables and each other were analyzed with Spearman’s rank correlation coefficients (*ρ*).

The chemokine CCL22 was identified as being significantly elevated in FEP patients compared to controls. After identifying potential confounding variables in bivariate analyses, we used a general linear model (GLM) to examine whether a psychotic disorder remains associated with elevated CCL22 even after adjusting for confounding variables. We explored the relationship of CCL22 with the other cytokines and chemokines by defining high CCL22 as being above the median of the FEP patient group, and investigating its association with other cytokines and chemokines, and their stability at the one-year follow-up using the Wilcoxon two sample test. These analyses were generated using SAS software, Version 9.3 of the SAS System for Windows. In addition, we visualized the correlations between cyto- and chemokines in a network. Pearson’s correlations and their significance were calculated between log-transformed cyto- and chemokine levels, separately for controls and FEP patients, using package Hmisc v4.2-0 (function rcorr)^36^ in the software environment R 3.6.1^37^. Undirected correlation networks were created in R with the igraph package v1.2.4 (function make_undirected_graph)^38^ and exported to Cytoscape v3.7.1^39^ for further editing using the RCy3 package^40^.

### Statistical analyses of PET data

One FEP patient with LAB genotype was excluded from the statistical analyses. Differences of *V*_T_ between PET study groups were tested with a repeated measures analysis of variance (rANOVA) model using TSPO binding genotype as a covariate. Since both CCL17 and CCL22 act through the same CCR4 receptor^41^, we used two rANOVA models to further test whether the mean of combined standardized CCL17 and CCL22 levels are associated with [^11^C]PBR28 *V*_T_ using all 17 VOIs as within-subject factors and binding status with and without group status as between-subjects factors. Statistical testing of PET data was done with IBM SPSS Statistics 25 software (IBM corp. Armonk, NY, USA) and GraphPad Prism 8.0.2 (GraphPad Software, San Diego, CA, USA). Detailed descriptions of the PET and MRI methods and the relevant statistical analyses can be found in the Supplementary Methods.

## Results

### Descriptive and clinical characteristics

The median age of both FEP patients and controls was 25.0 years. There were significantly more men in the patient than in the control group. The groups did not differ significantly in BMI or past-year cannabis use, but FEP patients had fewer years of education and smoked tobacco more often than controls. The antipsychotics most commonly used by the FEP patients were olanzapine and risperidone, while 12.4% were not using antipsychotics at baseline (Table 2).

**Table 2:** Baseline descriptive information on the study samples.

	Patients (*N* = 129)	Controls (*N* = 130)	*p*-Value^a^
Age, years, Md (Q1, Q3)	25.0 (22.0, 30.1)	25.0 (22.5, 31.0)	0.28
Male/female, *n*	82/47	59/71	**0.003**
Years of education, Md (Q1, Q3)	13 (12, 15.5)	15.5 (13, 17)	**<0.0001**
BMI, Md (Q1, Q3)	23.0 (21.3, 27.1)	24.0 (21.8, 26.9)	0.24
Current smoking, *n* (%)	28 (27.7 %)^b^	11 (9.1 %)^c^	**0.0003**
Cannabis use (past 12 months), *n* (%)	29 (26.1 %)^d^	25 (19.8 %)^e^	0.25
Antipsychotics used at baseline:			
Olanzapine, *n* (%)	45 (34.9 %)	0	
Risperidone, *n* (%)	45 (34.9 %)	0	
Quetiapine, *n* (%)	25 (19.4 %)	0	
Aripiprazole, *n* (%)	7 (5.4 %)	0	
Ziprasidone, *n* (%)	1 (0.8 %)	0	
Sertindole, *n* (%)	1 (0.8 %)	0	
Paliperidone, *n* (%)	2 (1.6 %)	0	
Haloperidol, *n* (%)	4 (3.1 %)	0	
Perphenazine, *n* (%)	3 (2.3 %)	0	
Chlorpromazine, *n* (%)	2 (1.6 %)	0	
No antipsychotic, *n* (%)	16 (12.4 %)	130 (100 %)	

^a^*χ*^2^ test used for categorical variables, Wilcoxon two-sample test with *t* approximation for continuous variables.

^b^Information missing on 28 participants.

^c^Information missing on 9 participants.

^d^Information missing on 18 participants.

^e^Information missing on 4 participants.

*Md* median, *Q1* first quartile, *Q3* third quartile.

Bold values indicate *p* < 0.05.

There were no major differences in demographic or PET imaging characteristics between the PET study groups. However, the PET FEP patients smoked tobacco more often and were on average 5 years younger than the HCs. The PET FEP patients had an average duration of illness of 4 months. Full baseline characteristics of the HEPS and TEPS samples are reported separately in the Supplementary Tables 3 and 4. Individual clinical and imaging information of FEP PET subjects can be found in Supplementary Table 5.

### Differences in serum cytokine and chemokine levels

The chemokine CCL22 level was significantly higher in FEP patients than in controls (*p* < .0001; Cohen’s *d* = 0.696). While the exact *p*-value for the Wilcoxon test could not be calculated, a *t* test with Sattertwaite correction for unequal variances gave a *p*-value <0.0001 for the difference in serum CCL22 levels between patients and controls. After Bonferroni correction for multiple testing (38 cyto- and chemokines), this *p*-value remained significant (*p* < 0.0001). In addition, TNF-α was higher in patients than in controls (*p* = 0.019), but the difference was not significant after correction for multiple testing. Since elevated serum CCL22 level was the only robust difference between patients and controls (Table 1), we focused on further characterizing this finding. CCL22 was significantly elevated both in the HEPS (*p* < 0.0001) and in the TEPS (*p* < 0.0001) samples. When the patients and controls from the HEPS who had been included in our previous report were excluded^17^, the difference in CCL22 level between the remaining 48 patients and 32 controls from the HEPS remained significant (*p* = 0.0015).

### Effects of potential confounding variables on serum CCL22 levels

Serum CCL22 level was associated with obesity, smoking, and olanzapine use, but not with sex, cannabis use, or other antipsychotic use in patients (Supplementary Table 6). It did not correlate significantly with age (*ρ* = −0.10, *p* = 0.11), sample storage time (*ρ* = −0.01, *p* 0.88), or chlorpromazine equivalent dose in patients (*ρ* = 0.10, *p* = 0.28).

We used a general linear model with CCL22 level as the dependent variable to adjust for the effects of smoking, obesity, and olanzapine use, and in addition included a covariate for study site (HEPS or TEPS). In this model (adjusted *R*^2^ = 0.22) FEP remained a significant predictor of elevated CCL22 (*β* = 245.3, *p* = 0.0019), while also smoking (*β* = 308.0, *p* = 0.0004), olanzapine use (*β* = 209.7, *p* = 0.036) and obesity (*β* = 303.2, *p* = 0.0042) were associated with CCL22 levels. A log transformation made CCL22 distributions somewhat more normal (Shapiro-Wilk *W* = 0.92 before, and 0.98 after), but this did not alter findings. After excluding patients and controls from our previous report^17^, FEP remained a significant predictor of elevated CCL22 (*β* = 297.3, *p* = 0.0017).

### Cyto- and chemokine network in FEP patients and controls

**Fig. 1:**
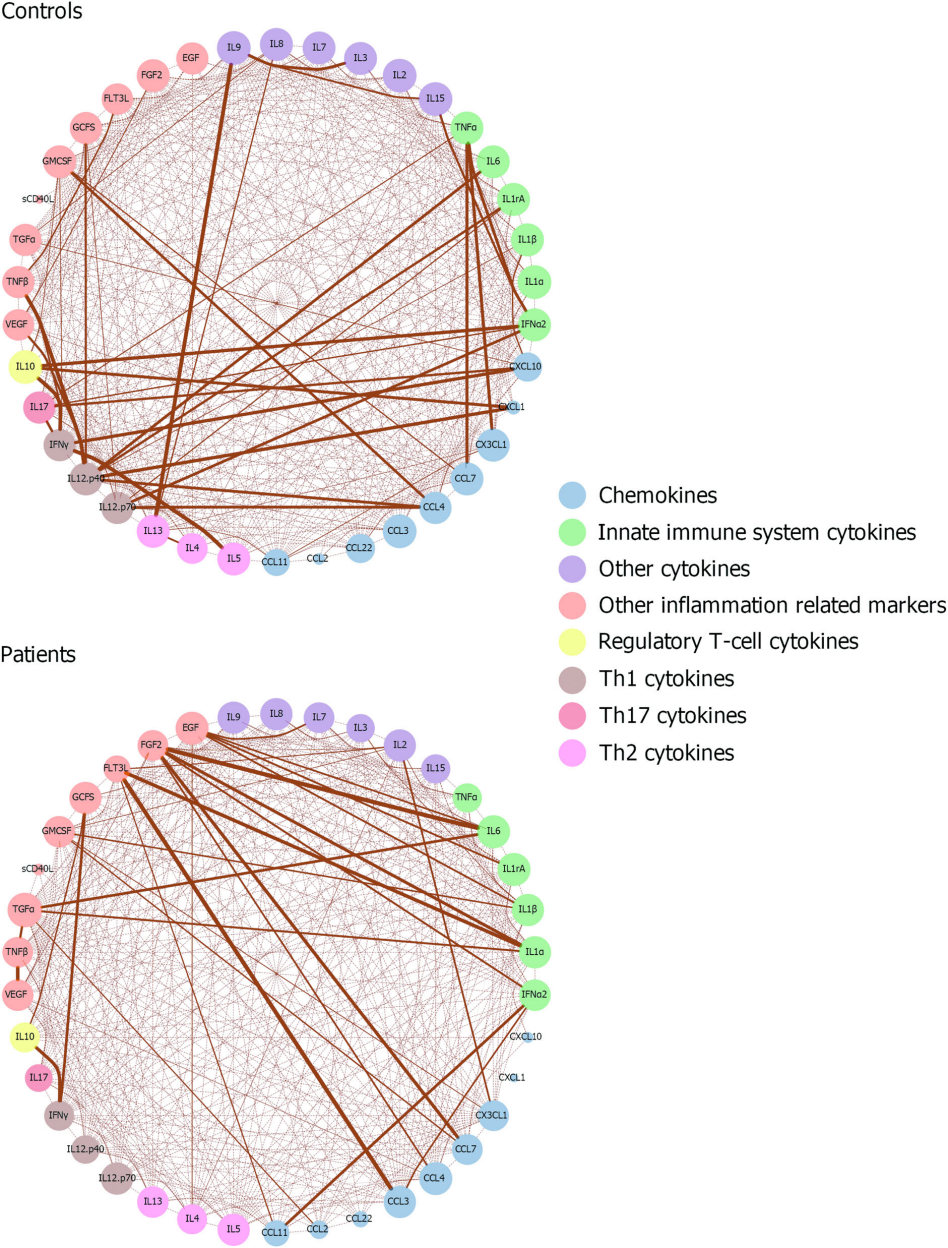
Spearman’s rank correlation coefficient networks of cyto- and chemokines in patients and controls. Correlations which are statistically significant after Bonferroni correction are presented. The strength of the line connecting two nodes (circles) reflects the degree of correlation, and the size of node for each cyto-and chemokine reflect the number and strength of correlations they have with other cyto- and chemokines. The nodes are grouped by color to structurally or functionally similar signaling molecules.

**Fig. 2:**
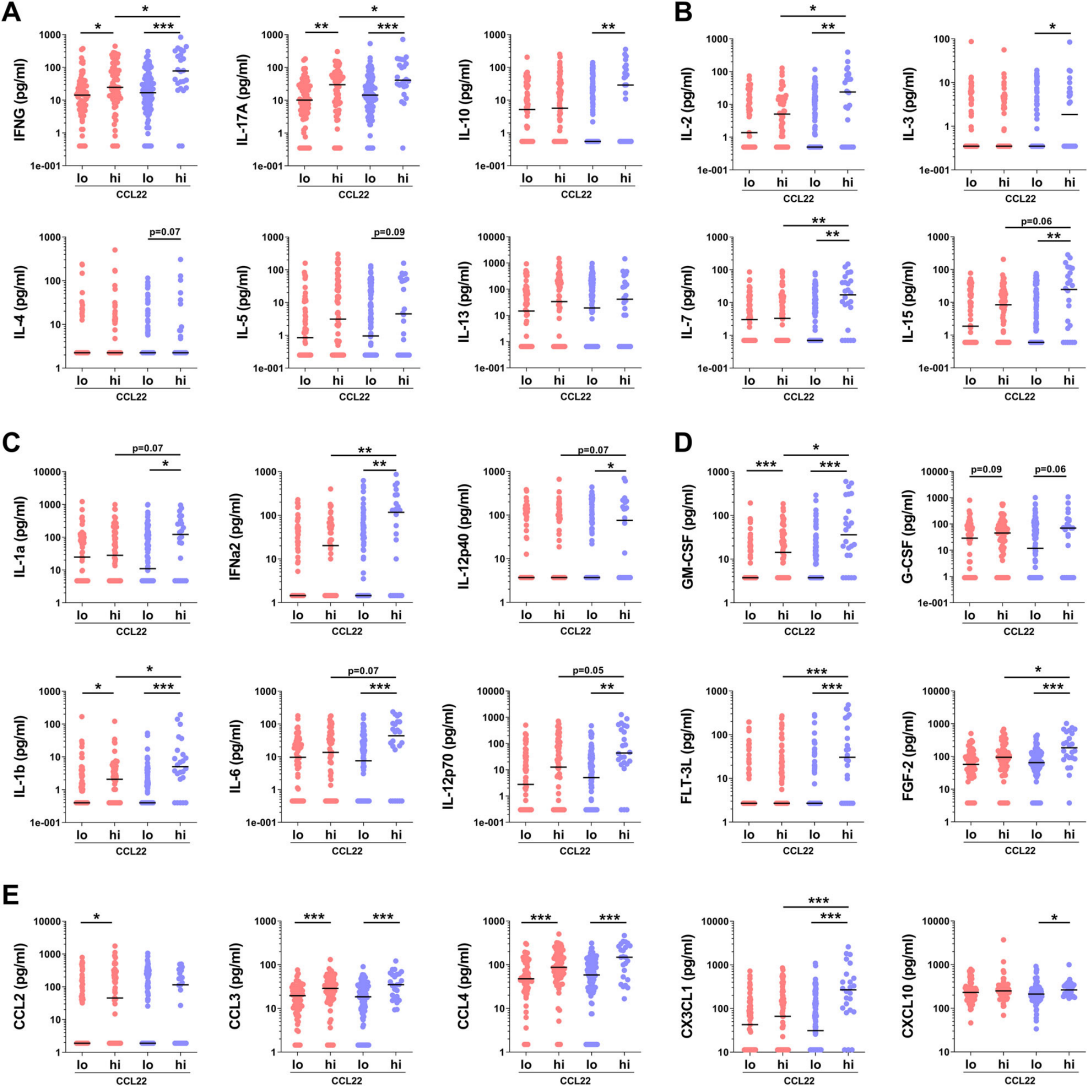
Cytokine, chemokine and growth factor concentration in FEP patients (red circles) and in control subjects with low or high CCL22. **a** Type cytokines of Th1, Th17 and Th2 cells and Tregs, **b** cytokines regulating T-cell activity and proliferation, **c** innate immunity cytokines, **d** growth factors and **e** chemokines. Black horizontal lines represents the median values. *Y*-axis show concentrations in logarithmic scale for visualization purposes. Linear concentration values were used to calculate p values using the Wilcoxon two sample test. *p < 0.05, **p < 0.01 and ***p < 0.001.

CCL22 levels showed more extensive correlations with other cyto- and chemokines in the controls than in the FEP patients (Supplementary Table 7). Fig. 1 presents the correlation network of cytokines and chemokines in FEP patients and in controls. Overall, the cytokine-chemokine network profile was somewhat different in FEP and control groups. In controls, T-cell cytokines of the adaptive immune system appeared as the most important nodes, whereas innate immunity cytokines and growth factors were the most important nodes in FEP patients. CCL22 levels showed a stronger correlation with Th1 (IFNg) and Th17 cytokines (IL-17) in FEP patients and controls, while marginal correlations with Th2 cytokines (IL-4, IL-5 and IL-13) were found in controls (Fig. 2 and Supplementary Tables 7, 8 and 9).

In order to further explore the possible contributors of CCL22 elevation and its consequences for the immune system, we defined high CCL22 as being the levels above the median of the FEP patient group (1277.5 pg/mL). In this analysis 49.6% of the FEP patients and 18.5 % of the controls belonged to the high CCL22 group (*χ*^2^ = 28.0, *p* < 0.0001). We then compared the distributions of other cytokines and chemokines separately in FEP patients and controls according to the high vs. low CCL22 classification. The levels of several cytokines, chemokines and growth factors were lower in high CCL22 FEP patients than in high CCL22 controls, namely CX3CL1, FGF-2, FLT-3L, IFN-a2, IL-1b, IL-7, IL-2, IL-12p70, IFN-g, and IL-17, suggesting a more restricted activation of the immune system despite of the high CCL22 levels in the patients (Fig. 2 and Supplementary Table 8).

### Longitudinal changes in cytokines and chemokines

One-year follow-up measurements were available for 58 FEP patients and 55 controls. CCL22 levels did not change significantly over time in patients or in controls. In FEP patients, there was a significant decrease in IFN-α2 (Wilcoxon Signed Rank *W* = 136, *p* = 0.013), IFN-γ (*W* = 270.5, *p* = 0.030) and IL-9 (*W* = 155.5, *p* = 0.042), and a significant increase in CCL2 (*W* = 256.5, *p* = 0.046). In controls, there was a significant decrease in TGF-α (*S* = − 292.5, *p* = 0.010). However, these differences were not significant after Bonferroni correction. When analyzing differences in the one-year follow-up measurements, CCL22 remained elevated in FEP patients vs. controls (Md 1097.7 vs. 843.7 pg/mL, *p* = 0.0007, surviving Bonferroni correction; Supplementary Table 10).

We further investigated whether belonging to the high CCL22 group was associated with persistently high CCL22 levels at one year follow-up in FEP patients, and whether the other cytokine and chemokine elevations seen in the high CCL22 group at baseline also persisted. CCL22 was significantly higher at one year follow-up in FEP patients with high baseline CCL22 compared to FEP patients with low baseline CCL22 (Md 1309.6 vs. 979.6 pg/mL, *p* = 0.0005), and a significant difference was still seen in seven other inflammatory markers; see Supplementary Table 11 for details.

### Clinical correlates of CCL22

FEP patients at baseline showed an association between serum CCL22 levels and hallucinations, conceptual disorganization, and worse verbal performance, but not with delusions, blunted affect, visuomotor performance or SOFAS (Supplementary Table 12). No significant correlations with cognitive functioning and SOFAS were seen in controls. Data on remission status at the one-year follow-up was available for 79 FEP patients, of whom 44 (55.7%) were in remission. In the follow-up, CCL22 level was 1080.2 pg/mL (Q1 884.9, Q3 1248.2) among those who were in remission and 1169.1 pg/mL (Q1 955.5, Q3 1565.9) in those who were not in remission (*p* = 0.16).

### PET studies—volume of interest analyses of [^11^C]PBR28 *V*_T_ group differences

**Fig. 3:**
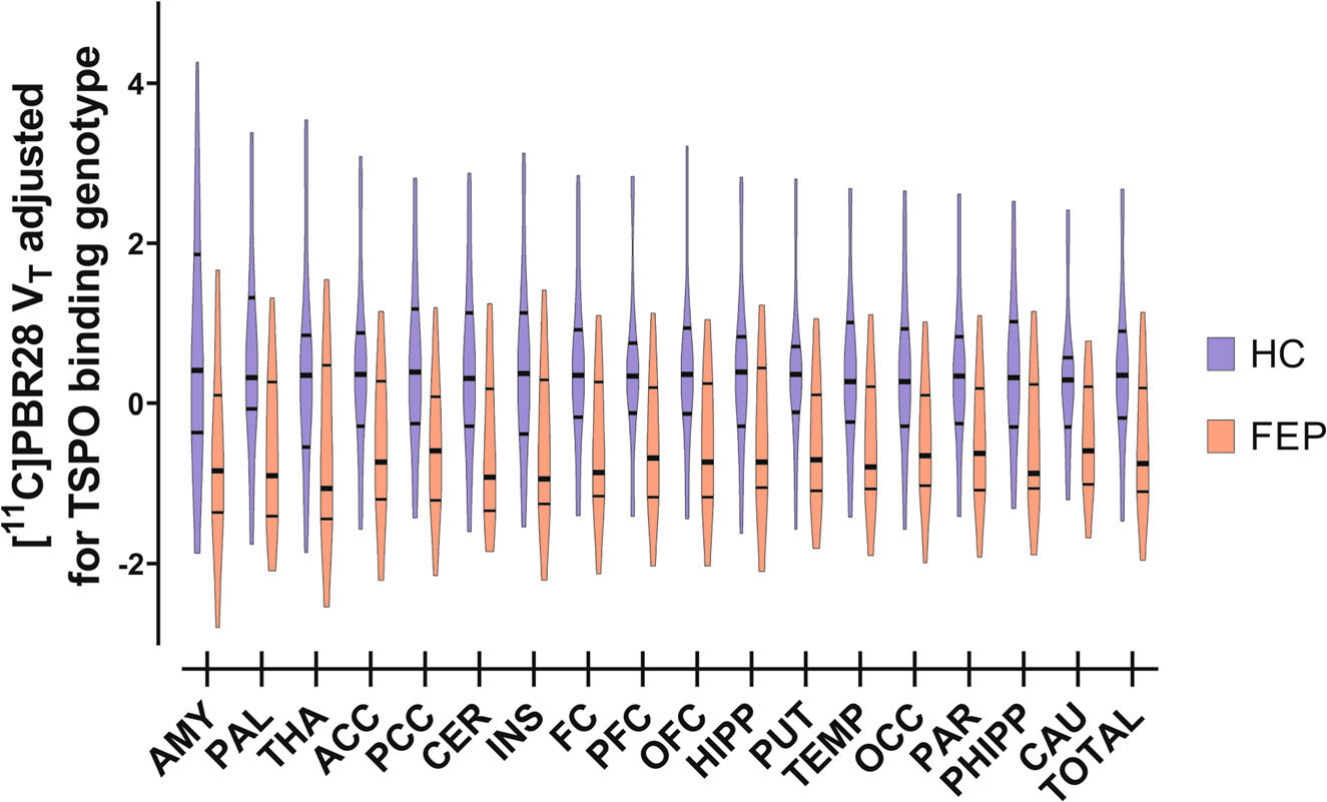
*V*_T_ of [^11^C]PBR28, adjusted for the effect of TSPO binding genotype, is lower in first episode psychosis patients (FEP, red bars, *n* = 13) than in healthy controls (HC, blue bars, *n* = 15) without significant differences between regions (df = 1, *F* = 5.635, *p* = 0.026; Cohen’s *d* = 0.936). The width of the figures represent the probability density at different values of adjusted *V*_T_ in 17 VOIs and their composite VOI. The VOIs are arranged in order of decreasing maximum mean of HC *V*_T_. Thick lines inside the figures indicate the median value, while the thin lines represent upper and lower quartiles. Abbreviations: ACC, anterior cingulate cortex; AMY, amygdala; CAU, caudate nucleus; CER, cerebellum; FC, frontal cortex; HIPP, hippocampus; INS, insula; OCC, occipital cortex; OFC, orbitofrontal cortex; PAR, parietal cortex; PCC, posterior cingulate cortex; PFC, prefrontal cortex; PHIPP, parahippocampal gyrus; PUT, putamen; TEMP, temporal cortex; THA, thalamus; TOTAL, composite VOI.

**Fig. 4:**
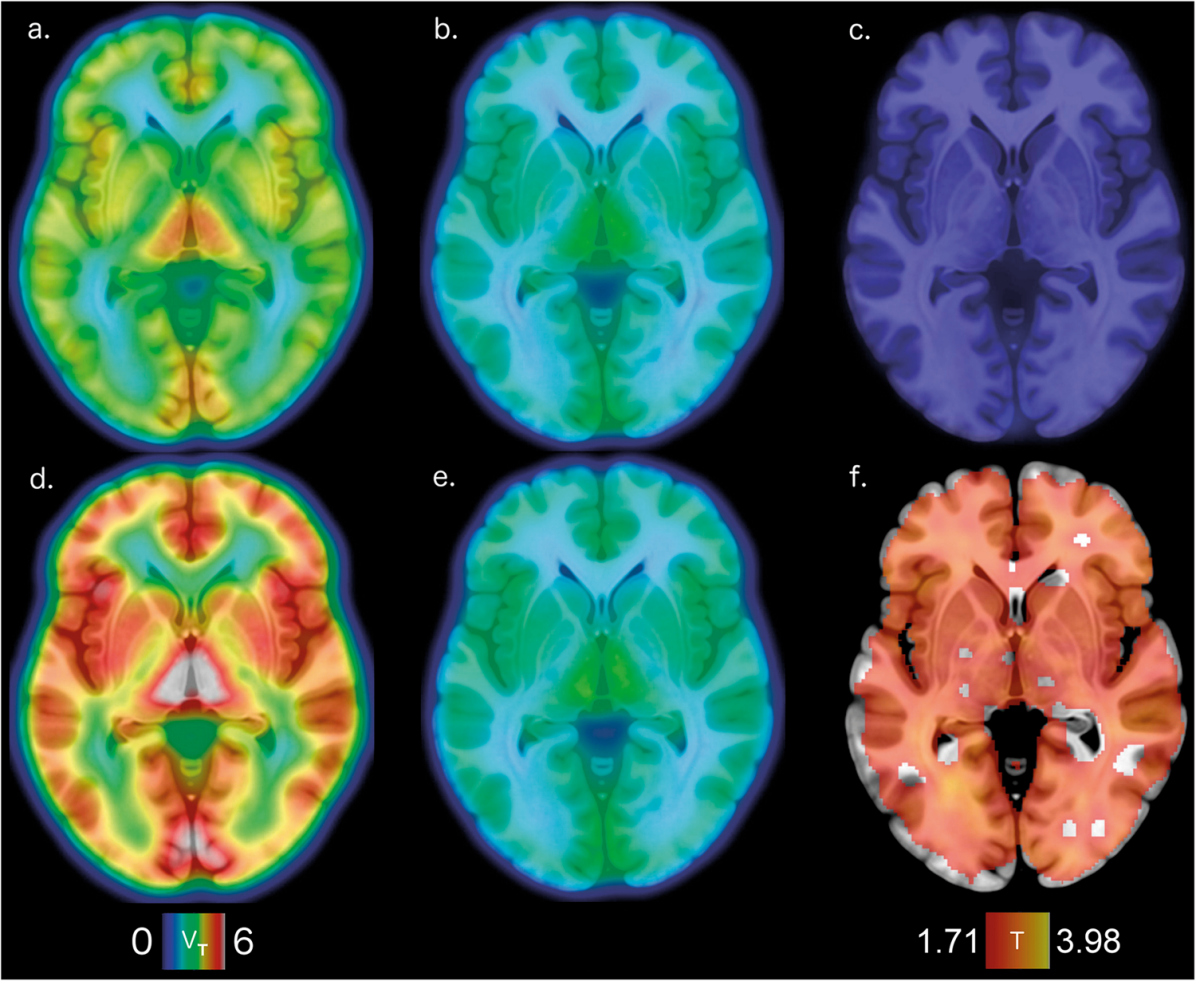
Voxel-wise tests of [^11^C]PBR28 V_T_. **a**–**e** Representative planes of mean [^11^C]PBR28 *V*_T_ in parametric images overlaid upon a anatomical reference template: **a** FEP HAB genotype subjects (*n*=8), **b** FEP MAB genotype subjects (*n*=5), **c** FEP LAB genotype subject (*n*=1), **d** HC HAB genotype subjects (*n*=9) and **e** HC MAB genotype subjects (*n*=6) separately. The color representation of *V*_T_, shown in the color bar on the lower left, is adjusted to to cover the variance between LAB, MAB, and HAB subjects in both groups. **f** A statistical parametric mapping (SPM) analysis shows one extensive cluster of lower *V*_T_ in FEPs (*n*=13) compared to HCs (*n*=15) visualized here with a red to yellow gradient representing voxel-wise T-score values. The lower limit of the color bar on the lower right shows the T-score height threshold and the upper limit denotes maximum peak value.

The groupwise characteristics of PET blood data can be found in Supplementary Figs. 1–3. The *V*_T_ of [^11^C]PBR28, covaried for the effect of TSPO binding genotype, was lower in FEP patients compared to HCs (*F*(1,25) = 5.635, *p* = 0.026; Cohen’s *d* = 0.936) without a significant group × region interaction (*F*(3.486) = 1.293, *p* = 0.281); Fig. 3 and Supplementary Fig. 4. The group effect was significant when including BMI (*F*(1,24) = 7.003, *p* = 0.014), or other previously reported possible confounding factors (*F*(1,21) = 4.655, *p* = 0.043) as covariates in the model^35^. Leaving out any one subject from the primary group analyses of [^11^C]PBR28 *V*_T_, covaried for the effect of TSPO binding genotype, did not change the interpretation of the test. Neither BPRS-E total (*p* = 0.789), positive (*p* = 0.810), negative (*p* = 0.696) or general symptom (*p* = 0.681) scores associated with [^11^C]PBR28 *V*_T_ in FEP patients. Parametric images of [^11^C]PBR28 *V*_T_ were used for an independent samples voxel-wise *t*-test of group differences when adjusting for the effect of TSPO binding genotype. We found one significant cluster of lower *V*_T_ in FEP patients compared to HCs (*p*_FWE-corr_ < 0.001, *k* > 400000); Fig. 4. Voxel-wise modeling of one FEP subject with LAB genotype shows that whole brain [^11^C]PBR28 *V*_T_ is globally <1; Fig. 4e.

### Associations of brain [^11^C]PBR28 *V*_T_ to serum CCL17 and CCL22 levels

**Fig. 5:**
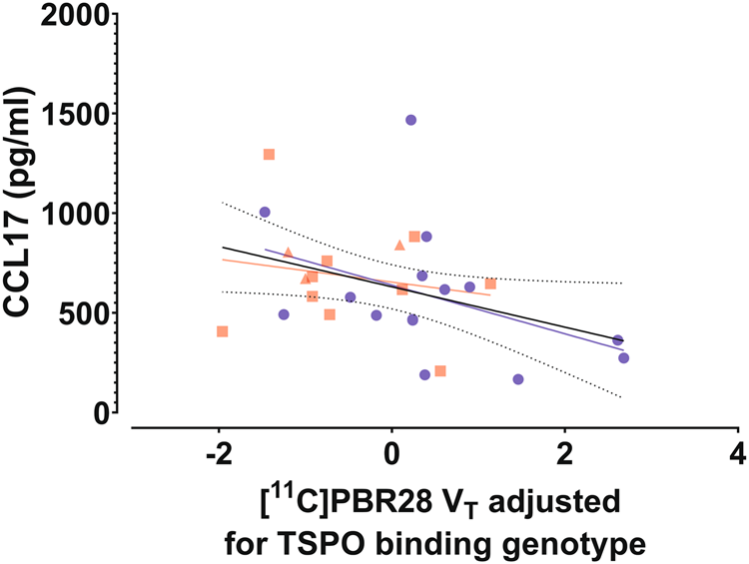
Association of brain [^11^C]PBR28 V_T_ to serum CCL17 level. Total GM VT of [^11^C]PBR28, adjusted for the effect of TSPO binding genotype, is shown plotted against peripheral blood serum concentrations of CCL17 in first episode psychosis patients (FEP; red squares for non-affective psychoses, *n* = 10; red triangles for affective psychoses, *n* = 3) and healthy controls (HC; blue squares, *n* = 15). A simple linear regression was used to test for association of adjusted *V*_T_ and CCL22 concentration in the whole sample, and within both groups separately. A statistically significant linear effect was found in the whole sample (*F*(1,26) = 4.361, *p* = 0.047), with an *R*^2^ of 0.144. The solid black line represents the linear regression line and the dotted black lines show the 95% confidence intervals of the fit. The best fit linear regression lines of the FEP and HC groups are shown as red and blue lines, respectively.

**Fig. 6:**
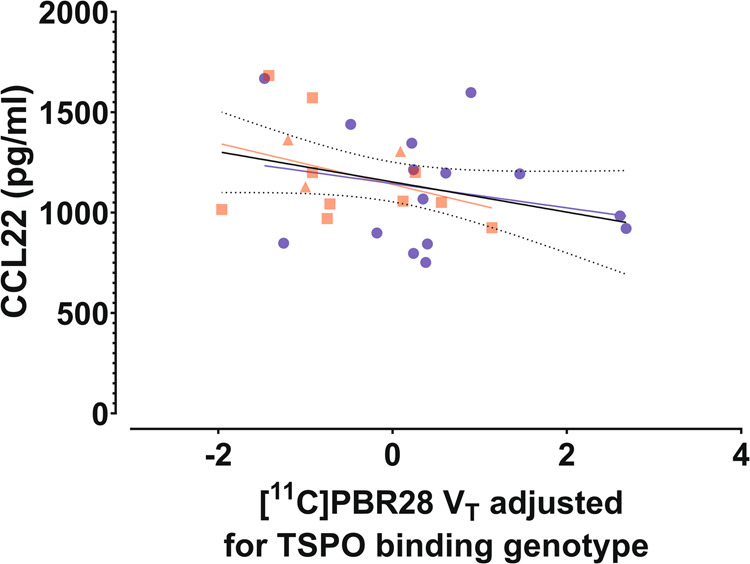
Association of brain [^11^C]PBR28 V_T_ to serum CCL22 level. Total GM VT of [^11^C]PBR28, adjusted for the effect of TSPO binding genotype, is shown plotted against peripheral blood serum concentrations of CCL22 in first episode psychosis patients (FEP; red squares for non-affective psychoses, *n* = 10; red triangles for affective psychoses, *n* = 3) and healthy controls (HC; blue squares, *n* = 15). A simple linear regression was used to test for association of adjusted *V*_T_ and CCL22 concentration in the whole sample, and both groups separately. A trend level statistically significant linear effect was found in the combined sample (*F*(1,26) = 3.017, *p* = 0.09), with an *R*^2^ of 0.104. The solid black line represents the linear regression line and the dotted black lines show the 95% confidence intervals of the fit. The best fit linear regression lines of the FEP and HC groups are shown as red and blue lines, respectively.

In the PET subgroup, there were no differences in the levels of CCR4 related chemokines, CCL22 or CCL17 between FEP patients and HCs (CCL22: *U* = 76.0, *p* = 0.322; CCL17: *U* = 67.5, *p* = 0.167; FEP CCL22: Md = 1128.5 pg/mL, Q1 = 1029.6, Q3 = 1333.6; FEP CCL17: Md = 672.4 pg/ml, Q1 = 537.0, Q3 = 823.1; HC CCL22: Md = 1067.7 pg/mL, Q1 = 847.9, Q3 = 1345.8; HC CCL17: Md = 491.3 pg/ml, Q1 = 362.6, Q3 = 685.4; see Supplementary Fig. 5). Linear regressions were calculated to predict total GM *V*_T_ based on CCL17 or CCL22 serum levels in the combined PET sample. For CCL17 a significant regression equation was found (*F*(1,26) = 4.361, *p* = 0.047), with an R^2^ of 0.144; Fig. 5. For CCL22 a trend level regression equation was found (*F*(1,26) = 3.017, *p* = 0.09), with an *R*^2^ of 0.104; Fig. 6. The correlation between serum levels of CCL17 and CCL22 was *ρ* = 0.498 (*p* = 0.008). In order to model the overall effect of CCR4 signaling, we standardized CCL17 and CCL22 levels and calculated their sum. The sum of standardized CCL17 and CCL22 levels were inversely associated to TSPO V_T_ in all 17 VOIs of the combined FEP and HC PET samples (*F*(1,25) = 4.898, *p* = 0.036) with a trend level association when taking into account the effect of group (*F*(1,24) = 3.767, *p* = 0.064). The association of chemokine levels to TSPO *V*_T_ remained significant when including age and smoking status as covariates in the model (*F*(1,23) = 4.402, *p* = 0.047).

## Discussion

The serum levels of chemokine CCL22 were higher in FEP patients at baseline and one year later in comparison to a matched general population control group. The baseline level of CCL22 was associated with more severe symptoms and worse performance in cognitive tests assessing verbal functioning. The *V*_T_ of [^11^C]PBR28, a biomarker for brain glial cell function, was lower in FEP patients compared to HCs. The levels of chemokines signaling via CCR4, CCL17 and CCL22, were associated with lower [^11^C]PBR28 *V*_T_ in the PET study of FEP patients and HCs.

### Chemokine and cytokine networks in first-episode psychosis

CCL22 was originally described as a macrophage-derived chemokine (MDC)^42^, and is produced by macrophages and dendritic cells (DCs)^43^. CCL22 and CCL17 are both ligands of the same chemokine receptor, C-C chemokine receptor type 4 (CCR4)^44^. CCR4 is expressed by type 2 helper T cells (Th2) and regulatory T cells (Tregs). Binding of CCL22 to CCR4 results in activation and regulation of T cell migration from lymph nodes to tissues^45^. Activation and functional differentiation of different Th cell subsets takes place in the lymph nodes after the engagement of the T-cell receptor (TCR) with the antigen-MHC class II complex on the surface of antigen presenting cells, DCs in particular^46^.

CCL22 is involved in many immunological disorders such as allergy, asthma, and atopic dermatitis through a Th2-mediated mechanism^47^, and it also participates in class switch recombination of immunoglobulins to IgE^48^. In some autoimmune diseases activation of Tregs by CCL22 has a protective effect^49,50^, but contrasting results have also been reported^51–54^. In addition, cancer cells and tumor-infiltrating macrophages express CCL22, which causes Treg migration into the tumor tissue, and suppression of an anti-tumor immune response, leading to worse prognosis and increased risk of metastasis^55–58^. CCL22 knockout mice have an enhanced T-cell response upon vaccination and stronger antitumor immunity, but are more susceptible to inflammatory conditions^45^. These data suggest that CCL22 signaling is critical in the maintenance of immunological homeostasis and control of immune-mediated diseases^45^.

Earlier cross-sectional studies suggest a role of CCR4 signaling in psychosis. Elevated CCL22 levels have been reported in patients with first-episode or chronic schizophrenia^59,60^, and one study also reported elevated levels of CCL17 in schizophrenia^60^. A recent study in FEP patients found elevated CCL17 but not CCL22 levels^61^. Furthermore, elevated CCL22 levels predicted relapse in schizophrenia patients^62^. However, CCL22 did not predict conversion to psychosis in a clinical study including high-risk individuals^63^. CCL22 and CCL17 have not been measured in many previous studies, which otherwise have analyzed an extensive battery of cytokines and chemokines^2,64,65^. An elevated CCL22 level has been reported also in autism and in frontotemporal dementia^66,67^, and CCL22 has also been suggested as a biomarker for medication response in major depressive disorder^68^. In multiple sclerosis (MS) CCL17, CCL22 and their receptor CCR4 have been studied extensively^69^. CCL22 has been suggested to be secreted by alternatively activated anti-inflammatory M2 microglia in the brain, and such cells have been shown to be present in MS lesions^70^. Elevated cerebrospinal fluid (CSF) levels of CCL22 and CCL17 have been reported in MS^69^, and the CSF levels of CCL22 have predicted the emergence of new MS lesions^71^. The evidence is consistent for the involvement of CCL17, CCL22 and CCR4 in the experimental autoimmune encephalomyelitis model of MS^72–74^. Altogether, the CCL17-CCL22-CCR4 chemokine–receptor axis is considered as a potential novel target for the treatment and prevention of CNS autoimmune diseases^69^.

To understand the functional role of CCR4, CCR4 and CCL17 knockout mice have been generated^75^. CCR4 knockout mice show diminished locomotor activity, exploratory behavior, and social exploration, while CCL17 knockout mice do not show altered behavior^75^. The authors concluded that there is a redundancy between CCL17 and CCL22, or CCL22 is the main activator of CCR4 in these behavioral influences^75^. A recent study indicates that mouse hippocampal neurons express CCL17, and CCL17 affects microglial morphology and synaptic transmission in the CA1 region, which suggests a neuronal origin of CCR4 signaling relevant for neuroinflammation^76^.

The levels of IFN-γ and IL-17 were higher in the high CCL22 controls compared with low CCL22 controls, or high CCL22 FEP patients. Surprisingly, the levels of Th2-type cytokines IL-4, IL-5 and IL-13 were comparable in high and low CCL22 subgroups of both patients and controls. High CCL22 controls had significantly elevated levels of anti-inflammatory IL-10 compared with low CCL22 controls. This indicates a simultaneous activation of effector, Th1, Th17, and regulatory T cell pathways in the controls with high CCL22 levels. Instead, no such difference between high versus low CCL22 subgroups was observed in the FEP patients. Furthermore, the high CCL22 controls had significantly higher levels of cytokines IFN-α2, IL-1b, IL-6, IL-12-p70, and the cytokine subunit IL-12p35 than low CCL22 controls, or patients with either low or high CCL22 levels. These cytokines are elementary in the activation and regulation of effector functions of Th1 (IFNs and IL-12) and Th17 (IL-1b and IL-6) cells, and they are secreted mainly by the cells of the innate immune system as a response to stimulation^77^. As the T-cell responses are tightly regulated by cells of the innate immune system, DCs in particular, it seems plausible that the observed alterations of effector and regulatory pathways of Th1 and Th17 are connected to an altered innate immune function in FEP. In conclusion, our data imply that the activation of CCL22 pathway in FEP patients is different from the activation of CCL22 pathway in the controls, and this implies differences in the primary stimulus driving CCL22 activation and/or in the signaling downstream of CCR4 between FEP patients and controls. The persistence of the observed immune marker profiles raises the question of whether these are consequences of early developmental events, as suggested by associations of neonatal immune system dysfunction with later life non-affective psychosis^78^.

Despite a relatively large sample size we did not find the previously reported elevations in IL-1β and IL-6 in FEP patients^79^. However, in line with a previous meta-analysis, TNF-α was higher in FEP patients compared to controls^48^. This may be due to differences in statistical analysis—we used nonparametric tests due to the highly skewed distributions, particularly of cytokines. Most of our patients were not antipsychotic-naïve, as patients were approached only when they were able to give informed consent. IL-1β and IL-6 have been shown to normalize with antipsychotic treatment^80^, which would be an understandable reason for not observing any respective elevations. However, differences in the control groups between studies are also possible. Our controls were recruited from the general population via the Population Register Centre, and only a lifetime history of psychotic disorder was used as an exclusion criterion. Consequently, subclinical depressive and anxiety symptoms were relatively common^81^, and there were no differences, for instance, in alcohol or cannabis use between patients and controls. While being matched with age, the gender distribution in our patient and control samples was not equal. However, sex was not associated to levels of CCL22 and consistently this difference did not affect our main findings.

### Brain glial cell activity in vivo and interaction with circulating chemokines

Consistent with a recent meta-analysis, our cohorts showed diminished brain glial cell function in FEP patients compared to controls^11^. The mean duration of psychosis in our sample was ~4 months, which suggests that factors associated with illness chronicity do not explain the effect. All participants were screened for active substance use, benzodiazepines shown to affect [^11^C]PBR28 binding^82^, and for active somatic illness. There were no significant differences of plasma tracer activity or active metabolite concentrations between groups, and the use of *V*_T_ as the [^11^C]PBR28 outcome measure cancels possible variation in blood tracer concentration. The high binding specificity of [^11^C]PBR28 to TSPO has previously been demonstrated in healthy participants, but not in FEP^33^. Here, the very low global *V*_T_ of the FEP participant with the LAB genotype indicates that psychosis does not associate with significant non-specific binding. The FEP participants were on average slightly younger than the HCs, but there was no associations between TSPO *V*_T_ and age. Addressing the previously reported confounders, namely age, sex and BMI, with relatively small effect sizes^35^, did not change the interpretation of our results.

Remarkably, studies using the first generation TSPO tracers and PET show an altogether higher index of brain glial cell function in FEP compared to controls^12^. While the results from the studies using the first generation TSPO tracer [^11^C]PK11195 are affected by a relatively low signal-to-noise ratio, and use of reference tissue modeling approaches despite ubiquitous brain TSPO expression, the second generation radioactive PET tracers, such as [^11^C]PBR28, offer very high selectivity to brain TSPO in non-human primates^13,14,33^. Therefore, the use of reference tissue models, chronic patient samples, and tracers with low signal-to-noise ratio might all contribute to the discrepancy between the results of first and second generation TSPO tracer studies. For example, TSPO expression may differ in chronic versus acute states, since TSPO expression shows dynamic longitudinal profiles within different glial cell lines after, for instance, lipopolysaccharide stimulation^9^. Only one previous study included FEP patients with a mean duration of illness under one year^83^. The antipsychotic naïve FEP patients in that study by Collste et al. had lower [^11^C]PBR28 *V*_T_ compared to controls, while a majority of the FEP subjects in the present study were using antipsychotic medications. However, there were no associations of [^11^C]PBR28 *V*_T_ to defined daily dose chlorpromazine equivalents in our FEP patient group. This is consistent with an absence of medication effects in previous second generation TSPO tracer studies^11^, and results from an animal study showing that haloperidol treatment at clinically relevant doses does not affect TSPO expression in rats^84^. Given the longitudinal variation of TSPO expression in various cell lines^9^, it is possible that differences in illness chronicity could explain some of the differences between previous studies.

The cellular origin of TSPO tracer selective binding has been under considerable debate recently^9,10^. While notions of TSPO overexpression associating to changes in functional state of microglia led to a view of TSPO expression as a marker of microglial immune activation^85^, further immunohistochemical studies in rats and humans have challenged this view^10^. TSPO markers co-localize to immunostaining of glial cells, such as microglia and astrocytes, but also to endothelial cells and neurons to some extent^16,86,87^. Constitutive TSPO expression in the endothelium is relatively low in the normal human brain^87^, and the relatively greater number of glial cells compared to endothelial cells within the human brain should affect the relative magnitude of endothelial and neuronal signal when measuring TSPO with PET on a macroanatomical scale. However, this does not entirely rule out effects due to pathological changes in, for example, vasculature TSPO expression^88^. Recent efforts to validate a kinetic model to take into account a putative irreversible endothelial TSPO binding compartment are still reliant on unproven theoretical assumptions, and do not account for illness effects, which muddles the interpretation of specific binding derived from this model^89^. Complicating the issue further, different TSPO antibodies have different selectivities for TSPO in different cell lines^87^. Also, TSPO immunoreactivity can present as elevated or decreased expression in astrocytes, microglial cells and vascular endothelial cells as a response to some immunological stimuli, but not necessarily others^16,87,90^. A recent study compared TSPO PET of schizophrenia patients and controls with combined peripheral and CSF measures of IL1β, IFNγ, IL-10, IL-6 and TNF-α. This study by Coughlin et al. showed that the lower TSPO PET signal in the brain of schizophrenia patients did not associate with cytokine levels in the plasma or CSF^65^. Further translational evaluation of these results by Coughlin et al., using a maternal immune activation mouse model of schizophrenia, suggested that there is no positive association of TSPO expression with central low-grade inflammation, and that downregulation of TSPO via non-immune mechanisms could predispose to low-grade inflammation via the reduction of the anti-inflammatory properties of TSPO^16,91^.

Our findings on associations of CCR4 related chemokines CCL17 and CCL22 to [^11^C]PBR28 *V*_T_ suggests that changes in CCR4 signaling are reflected in the function of glial cells, or vice versa. Peripheral metabolism and the immune environment during development present as possible mechanistic explanations to altered glial functioning as they are are poised to influence the function or number of glial cells during adulthood^92,93^. Our result of relatively low TSPO availability in FEP does not support the notion of elevated TSPO expression as a marker of central inflammation in psychosis, and together with the earlier studies by Notter et al.^16^, Collste et al.^83^ and Plavén-Sigray et al.^11^, suggest that the onset of psychosis could be either unrelated to neuroinflammation or associated with low-grade neuroinflammation manifesting as lower second generation TSPO tracer *V*_T_. Consistently, the pattern of the peripheral cytokine network associated with high CCL22 levels in the FEP patients does not demonstrate a robust activation of the inflammatory pathways in a similar way as seen in the healthy controls with high CCL22 levels. Further studies are needed to characterize the relevance of high circulating CCL22 levels or enhanced CCR4 signaling for CNS function, as well as for the altered cyto/chemokine networks in psychotic disorders.

## Conclusions

Serum levels of chemokine CCL22 are persistently elevated in early psychotic disorders. CCL22 levels are also associated with the core symptom domains of psychosis, and with altered patterns of adaptive/innate immunity signaling. Our results do not support the over-active microglia hypothesis of psychosis. However, brain glial cell function or density is lower in FEP, and inversely associated with serum levels of chemokine receptor CCR4 ligands CCL17 and CCL22. Our results indicate a longstanding dysregulation of peripheral immune function in early psychosis with behavioral correlates possibly mediated through cross-talk with dysfunctional or a decreased number of glial cells.

## Supplementary information

Supplementary Methods File

Supplementary Results File

## References

[1] Goldsmith DR, Rapaport MH, Miller BJ (2016). A meta-analysis of blood cytokine network alterations in psychiatric patients: comparisons between schizophrenia, bipolar disorder and depression. Mol. Psychiatry.

[2] Frydecka D (2018). Profiling inflammatory signatures of schizophrenia: a cross-sectional and meta-analysis study. Brain Behav. Immun..

[3] Wang AK, Miller BJ (2018). Meta-analysis of cerebrospinal fluid cytokine and tryptophan catabolite alterations in psychiatric patients: comparisons between schizophrenia, bipolar disorder, and depression. Schizophr. Bull..

[4] Volk DW (2017). Role of microglia disturbances and immune-related marker abnormalities in cortical circuitry dysfunction in schizophrenia. Neurobiol. Dis..

[5] Fillman SG (2016). Elevated peripheral cytokines characterize a subgroup of people with schizophrenia displaying poor verbal fluency and reduced Broca’s area volume. Mol. Psychiatry.

[6] Boerrigter D (2017). Using blood cytokine measures to define high inflammatory biotype of schizophrenia and schizoaffective disorder. J. Neuroinflammation.

[7] Monji A, Kato T, Kanba S (2009). Cytokines and schizophrenia: microglia hypothesis of schizophrenia. Psychiatry Clin. Neurosci..

[8] Smith RS (1992). A comprehensive macrophage-T-lymphocyte theory of schizophrenia. Med. Hypotheses.

[9] Guilarte TR (2019). TSPO in diverse CNS pathologies and psychiatric disease: a critical review and a way forward. Pharmacol. Ther..

[10] Notter T, Coughlin JM, Sawa A, Meyer U (2018). Reconceptualization of translocator protein as a biomarker of neuroinflammation in psychiatry. Mol. Psychiatry.

[11] Plavén-Sigray P (2018). Positron emission tomography studies of the glial cell marker translocator protein in patients with psychosis: a meta-analysis using individual participant data. Biol. Psychiatry.

[12] Marques TR (2019). Neuroinflammation in schizophrenia: meta-analysis of in vivo microglial imaging studies. Psychol. Med..

[13] Imaizumi M (2008). Brain and whole-body imaging in nonhuman primates of [11 C]PBR28, a promising PET radioligand for peripheral benzodiazepine receptors. Neuroimage.

[14] Li F (2016). Translocator protein 18 kDa (TSPO): an old protein with new functions?. Biochemistry.

[15] Gandal MJ (2018). Transcriptome-wide isoform-level dysregulation in ASD, schizophrenia, and bipolar disorder. Science.

[16] Notter T (2018). Translational evaluation of translocator protein as a marker of neuroinflammation in schizophrenia. Mol. Psychiatry.

[17] Mäntylä T (2015). Altered activation of innate immunity associates with white matter volume and diffusion in first-episode psychosis. PLoS ONE.

[18] Kori-Lindner C (2013). World medical association declaration of Helsinki. JAMA.

[19] Larsson A (2015). The effects of age and gender on plasma levels of 63 cytokines. J. Immunol. Methods.

[20] Unamuno X (2018). Adipokine dysregulation and adipose tissue inflammation in human obesity. Eur. J. Clin. Invest..

[21] Shiels MS (2014). Cigarette smoking and variations in systemic immune and inflammation markers. J. Natl. Cancer Inst..

[22] Karoly HC, Bidwell LC, Mueller RL, Hutchison KE (2018). Investigating the relationships between alcohol consumption, cannabis use, and circulating cytokines: a preliminary analysis. Alcohol Clin. Exp. Res.

[23] Hardikar S (2014). Intraindividual variability over time in plasma biomarkers of inflammation and effects of long-term storage. Cancer Causes Control.

[24] Mantere O (2019). Immunomodulatory effects of antipsychotic treatment on gene expression in first-episode psychosis. J. Psychiatr. Res..

[25] Andreasen NC (1989). The scale for the assessment of negative symptoms (SANS): conceptual and theoretical foundations. Br. J. Psychiatry Suppl..

[26] Ventura J. et al. Brief psychiatric rating scale expanded version 4.0: scales anchor points and administration manual (1993).

[27] Kay SR, Fiszbein A, Opler LA (1987). The positive and negative syndrome scale (PANSS) for schizophrenia. Schizophr. Bull..

[28] Andreasen NC (2005). Remission in schizophrenia: proposed criteria and rationale for consensus. Am. J. Psychiatry.

[29] American Psychiatric Association. *Diagnostic and Statistical Manual of Mental Disorders*. 4th ed (American Psychiatric Association, Washington, DC, 1994).

[30] Hillmer AT (2017). Microglial depletion and activation: a [11C]PBR28 PET study in nonhuman primates. EJNMMI Res.

[31] Yoder KK (2013). Influence of TSPO Genotype on 11C-PBR28 Standardized Uptake Values. J. Nucl. Med..

[32] Kreisl WC (2013). A genetic polymorphism for translocator protein 18 Kda affects both in vitro and in vivo radioligand binding in human brain to this putative biomarker of neuroinflammation. J. Cereb. Blood Flow. Metab..

[33] Fujita M (2008). Kinetic analysis in healthy humans of a novel positron emission tomography radioligand to image the peripheral benzodiazepine receptor, a potential biomarker for inflammation. Neuroimage.

[34] Collste K (2016). Test–retest reproducibility of [11C]PBR28 binding to TSPO in healthy control subjects. Eur. J. Nucl. Med Mol. Imaging.

[35] Tuisku J (2019). Effects of age, BMI and sex on the glial cell marker TSPO — a multicentre [11C]PBR28 HRRT PET study. Eur. J. Nucl. Med Mol. Imaging.

[36] Frank E. Harrell, Jr. Hmisc: Harrell Miscellaneous. R package version 4.2-0 (2019) https://cran.r-project.org/package=Hmisc.

[37] R Core Team. R: a language and environment for statistical computing. (2013) http://www.r-project.org/.

[38] Csardi G, Nepusz T (2006). The igraph software package for complex network research. InterJournal Complex Syst..

[39] Shannon P (2003). Cytoscape: a software environment for integrated models of biomolecular interaction networks. Genome Res..

[40] Gustavsen JA, Pai S, Isserlin R, Demchak B, Pico AR (2019). RCy3: network biology using cytoscape from within R. F1000Research.

[41] Yoshie O, Matsushima K (2015). CCR4 and its ligands: from bench to bedside. Int Immunol..

[42] Mantovani A, Gray PA, Van Damme J, Sozzani S (2000). Macrophage-derived chemokine (MDC). J. Leukoc. Biol..

[43] Vulcano M (2001). Dendritic cells as a major source of macrophage-derived chemokine/CCL22 in vitro and in vivo. Eur. J. Immunol..

[44] Nomiyama H, Osada N, Yoshie O (2013). Systematic classification of vertebrate chemokines based on conserved synteny and evolutionary history. Genes Cells.

[45] Rapp M (2019). CCL22 controls immunity by promoting regulatory T cell communication with dendritic cells in lymph nodes. J. Exp. Med..

[46] Jenkins MK (2001). In vivo activation of antigen-specific CD4 T cells. Annu. Rev. Immunol..

[47] Perros F, Hoogsteden HC, Coyle AJ, Lambrecht BN, Hammad H (2009). Blockade of CCR4 in a humanized model of asthma reveals a critical role for DC-derived CCL17 and CCL22 in attracting Th2 cells and inducing airway inflammation. Allergy.

[48] Zhang Y, Fear DJ, Willis-Owen SAG, Cookson WO, Moffatt MF (2016). Global gene regulation during activation of immunoglobulin class switching in human B cells. Sci. Rep..

[49] Bischoff L (2015). Cellular mechanisms of CCL22-mediated attenuation of autoimmune diabetes. J. Immunol..

[50] Eby JM (2015). CCL22 to activate Treg migration and suppress depigmentation in vitiligo. J. Invest. Dermatol..

[51] Ushio A (2018). CCL22-producing resident macrophages enhance T cell response in Sjögren’s syndrome. Front Immunol..

[52] Molineros JE (2017). Confirmation of five novel susceptibility loci for Systemic Lupus Erythematosus (SLE) and integrated network analysis of 82 SLE susceptibility loci. Hum. Mol. Genet..

[53] Rump L, Mattey DL, Kehoe O, Middleton J (2017). An initial investigation into endothelial CC chemokine expression in the human rheumatoid synovium. Cytokine.

[54] Fujii H, Shimada Y, Hasegawa M, Takehara K, Sato S (2004). Serum levels of a Th1 chemoattractant IP-10 and Th2 chemoattractants, TARC and MDC, are elevated in patients with systemic sclerosis. J. Dermatol. Sci..

[55] Anz D (2015). Suppression of intratumoral CCL22 by type I interferon inhibits migration of regulatory T cells and blocks cancer progression. Cancer Res..

[56] Yeung OWH (2015). Alternatively activated (M2) macrophages promote tumour growth and invasiveness in hepatocellular carcinoma. J. Hepatol..

[57] Klarquist J (2016). Ccl22 diverts T regulatory cells and controls the growth of melanoma. Cancer Res..

[58] Wei Y (2017). C-C motif chemokine 22 ligand (CCL22) concentrations in sera of gastric cancer patients are related to peritoneal metastasis and predict recurrence within one year after radical gastrectomy. J. Surg. Res..

[59] Dimitrov DH (2013). Differential correlations between inflammatory cytokines and psychopathology in veterans with schizophrenia: Potential role for IL-17 pathway. Schizophr. Res..

[60] Hong S (2017). Abnormalities in chemokine levels in schizophrenia and their clinical correlates. Schizophr. Res..

[61] Malmqvist A (2019). Increased peripheral levels of TARC/CCL17 in first episode psychosis patients. Schizophr. Res..

[62] Schwarz E, Guest PC, Steiner J, Bogerts B, Bahn S (2012). Identification of blood-based molecular signatures for prediction of response and relapse in schizophrenia patients. Transl. Psychiatry.

[63] Perkins DO (2015). Towards a psychosis risk blood diagnostic for persons experiencing high-risk symptoms: preliminary results from the NAPLS project. Schizophr. Bull..

[64] Bocchio-Chiavetto L (2018). Immune and metabolic alterations in first episode psychosis (FEP) patients. Brain Behav. Immun..

[65] Coughlin JM (2016). In vivo markers of inflammatory response in recent-onset schizophrenia: a combined study using [11C]DPA-713 PET and analysis of CSF and plasma. Transl. Psychiatry.

[66] Roos P (2018). Inflammatory markers of CHMP2B-mediated frontotemporal dementia. J. Neuroimmunol..

[67] AL-Ayadhi LY, Mostafa GA (2013). Elevated serum levels of macrophage-derived chemokine and thymus and activation-regulated chemokine in autistic children. J. Neuroinflammation.

[68] Milenkovic VM (2017). Macrophage-derived chemokine: a putative marker of pharmacological therapy response in major depression?. Neuroimmunomodulation.

[69] Scheu S, Ali S, Ruland C, Arolt V, Alferink J (2017). The C-C chemokines CCL17 and CCL22 and their receptor CCR4 in CNS autoimmunity. Int J. Mol. Sci..

[70] Peferoen LAN (2015). Activation status of human microglia is dependent on lesion formation stage and remyelination in multiple sclerosis. J. Neuropathol. Exp. Neurol..

[71] Håkansson I (2018). Neurofilament levels, disease activity and brain volume during follow-up in multiple sclerosis. J. Neuroinflammation.

[72] Forde EA, Dogan R-NE, Karpus WJ (2011). CCR4 contributes to the pathogenesis of experimental autoimmune encephalomyelitis by regulating inflammatory macrophage function. J. Neuroimmunol..

[73] Poppensieker K (2012). CC chemokine receptor 4 is required for experimental autoimmune encephalomyelitis by regulating GM-CSF and IL-23 production in dendritic cells. Proc. Natl. Acad. Sci. USA.

[74] Jordão MJC (2019). Single-cell profiling identifies myeloid cell subsets with distinct fates during neuroinflammation. Science.

[75] Ambrée O (2016). Reduced locomotor activity and exploratory behavior in CC chemokine receptor 4 deficient mice. Behav. Brain Res.

[76] Fülle L (2018). CCL17 exerts a neuroimmune modulatory function and is expressed in hippocampal neurons. Glia.

[77] Mogensen TH (2009). Pathogen recognition and inflammatory signaling in innate immune defenses. Clin. Microbiol Rev..

[78] Gardner RM, Dalman C, Wicks S, Lee BK, Karlsson H (2013). Neonatal levels of acute phase proteins and later risk of non-affective psychosis. Transl. Psychiatry.

[79] Upthegrove R, Manzanares-Teson N, Barnes NM (2014). Cytokine function in medication-naive first episode psychosis: a systematic review and meta-analysis. Schizophr. Res..

[80] Miller BJ, Buckley P, Seabolt W, Mellor A, Kirkpatrick B (2011). Meta-analysis of cytokine alterations in schizophrenia: clinical status and antipsychotic effects. Biol. Psychiatry.

[81] Lindgren M (2017). Childhood adversities and clinical symptomatology in first-episode psychosis. Psychiatry Res..

[82] Kalk NJ (2013). Are prescribed benzodiazepines likely to affect the availability of the 18 kDa translocator protein (TSPO) in PET studies?. Synapse.

[83] Collste K (2017). Lower levels of the glial cell marker TSPO in drug-naive first-episode psychosis patients as measured using PET and [11C]PBR28. Mol. Psychiatry.

[84] Bloomfield PS (2018). The effects of haloperidol on microglial morphology and translocator protein levels: an in vivo study in rats using an automated cell evaluation pipeline. J. Psychopharmacol..

[85] Venneti S, Lopresti BJ, Wiley CA (2006). The peripheral benzodiazepine receptor (Translocator protein 18kDa) in microglia: From pathology to imaging. Prog. Neurobiol..

[86] Betlazar C, Harrison-Brown M, Middleton R, Banati R, Liu G-J (2018). Cellular sources and regional variations in the expression of the neuroinflammatory marker translocator protein (TSPO) in the normal brain. Int J. Mol. Sci..

[87] Cosenza-Nashat M (2009). Expression of the translocator protein of 18 kDa by microglia, macrophages and astrocytes based on immunohistochemical localization in abnormal human brain. Neuropathol. Appl. Neurobiol..

[88] Bahney J, von Bartheld CS (2018). The cellular composition and glia–neuron ratio in the spinal cord of a human and a nonhuman primate: comparison with other species and brain regions. Anat. Rec..

[89] Veronese M (2018). Kinetic modelling of [11 C]PBR28 for 18 kDa translocator protein PET data: a validation study of vascular modelling in the brain using XBD173 and tissue analysis. J. Cereb. Blood Flow. Metab..

[90] Pannell M (2019). Imaging of translocator protein upregulation is selective for pro-inflammatory polarized astrocytes and microglia. Glia.

[91] Bae KR, Shim HJ, Balu D, Kim SR, Yu SW (2014). Translocator protein 18 kDa negatively regulates inflammation in microglia. J. Neuroimmune Pharmacol..

[92] Erny D (2015). Host microbiota constantly control maturation and function of microglia in the CNS. Nat. Neurosci..

[93] Baufeld C, Osterloh A, Prokop S, Miller KR, Heppner FL (2016). High-fat diet-induced brain region-specific phenotypic spectrum of CNS resident microglia. Acta Neuropathol..

