# Supplementary material for: Elevated serum chemokine CCL22 levels in first-episode psychosis: associations with symptoms, peripheral immune state and in vivo brain glial cell function
Source: Transl Psychiatry. 2020 Mar 16;10:94. doi: 10.1038/s41398-020-0776-z (PMC7075957; doi:10.1038/s41398-020-0776-z)
Supplement: Supplementary file 1 — Supplementary Methods File [file 41398_2020_776_MOESM1_ESM.docx]

Supplementary Methods File

Subject recruitment and inclusion/exclusion criteria

For recruitment to the Helsinki Early Psychosis Study (HEPS) sample, psychosis was defined as a score of at least 4 in the items assessing unusual thought content (delusions) or hallucinations in the Brief Psychiatric Rating Scale, Expanded version 4.0 (BPRS-E) (1). The diagnoses were later verified using the research version of the Structured Clinical Interview for DSM-IV (2) complemented by a review of all medical records by a senior psychiatrist (JS). In the Turku Early Psychosis Study (TEPS) psychotic disorders as defined by the structured clinical interview for DSM-IV axis I disorders (SCID-I/NP), complemented by a review of all medical records, were included, except for substance induced psychoses and psychoses due to general medical condition (2). In both sites controls, matched by age, sex and region of residence, were identified from the Population Register Center and sent an invitation letter to participate in the study. Psychotic disorders and chronic neurological or endocrinological diseases were exclusion criteria for the controls. For analyzing the correlation of CCL22 levels with symptoms, we assessed the severity of key symptoms of psychosis as follows: severity of delusions was assessed using P1 Delusions in PANSS, considered to correspond to item 11 Unusual thought content in BPRS-E; severity of hallucinations was assessed using PANSS P3 Hallucinatory behavior or BPRS-E item 10 Hallucinations, disorganization (positive formal thought disorder) was assessed using PANSS P2 Conceptual disorganization or BPRS-E item 15 Conceptual disorganization, and blunted affect using PANSS N1 Blunted Affect or BPRS-E item 16 Blunted affect.

In the PET study sample the healthy individuals were recruited from the national population registry, local educational institutions, and by local newspaper advertisement. Three healthy controls were ruled out from statistical tests due to technical failure resulting in incomplete blood data, and one FEP was ruled out due to aborted PET scan. Overall, the study sample for statistical tests of [^11^C]PBR28 V_T_ consisted of 14 FEPs and 15 HCs. The somatic status of all the PET subjects was confirmed by medical examination, blood and urine tests, electrocardiography, SCID-I/NP (2), and the Structured interview for prodromal syndromes (SIPS version 5.0) (3). Pregnancy was ruled out by urine and/or blood screening. Subjects with a chronic medical or neurological condition affecting the brain, history of head trauma with loss of consciousness, and neurodevelopmental disorders were excluded. One FEP subject had been diagnosed with juvenile idiopatic arthritis, but had been free of symptoms and need of medication. Lifetime substance use was documented, and current use was controlled with a urine screen prior to the PET scan. Subjects with a lifetime DSM-IV Axis I diagnosis, substance dependency, or who had used any illicit substances two months prior to scanning, were excluded. Alcohol use was assessed using the alcohol use disorders identification test and tobacco use was documented using a questionnaire adaptation of the questionnaire recommended by the World Health Organisation (WHO, 1995). Illegal substance use was documented based on a previously described questionnaire (4). All PET subjects restrained from substance use two months prior to PET and substance use was screened using a urine toxicology test (InstAlert; THC-COOH, 50 ng/ml cut off; amphetamine, 500ng/ml cut-off; buprenorphine, 5ng/ml cut-off; cocaine, 300ng/ml cut-off; methadone, 300ng/ml cut-off; opiates, 300ng/ml cut-off). The total duration of psychotic illness (DOI) was defined as the time between fulfillment of SIPS 5.0 psychotic syndrome criteria and PET scanning. Duration of untreated psychosis (DUP) was defined similarly as the time from presence of psychotic syndrome to the start date of adequate antipsychotic drug treatment. Benzodiazepine use was recorded and all subjects using diazepam were required to switch to using lorazepam after an adequate wash-out period (5).

Cognitive testing

Cognitive functioning was assessed with tests that had been included in the protocols in both sites, including measures from the Wechsler Adult Intelligence Scale, Wechsler Memory Scale, Trail Making Tests, and Verbal Fluency (Supplementary Table 11).

To summarize baseline cognitive performance, a confirmatory two-dimensional factor model of verbal and visuomotor performance was estimated with Mplus 8 (6) using maximum likelihood estimation with robust standard errors (MLR) and default settings, and factor scores for the two factors to be used in the analyses were estimated with the regression method. To correct their highly skewed distributions, Trails Making performance times were first inverted to speed (unit 1/s) and rescaled (× 1000); other cognitive variables were satisfactorily normal in their original form.

The cognitive factor fit was not optimal but serviceable, with Comparative Fit Index (CFI) value 0.94, Standardized Root Mean Square Residual (SRMR) value 0.05, and factor determinacy values 0.90 for the both factors. The two dimensions correlated at 0.81. Factor loadings are presented in Supplementary Table 12.

Supplementary Table 1. Cognitive tests

| Task | Variable used | Function |
| --- | --- | --- |
| Trail Making A (7) | 1/time to complete ^1^ | Visuomotor speed |
| Trail Making B (7) | 1/time to complete ^1^ | Executive functioning |
| WAIS-III Vocabulary (8) | Total score | General verbal ability |
| WAIS-III Digit Symbol (8) | Total score | Processing speed |
| Verbal Fluency, animals (9) | Number of correct words generated in 60 sec | Verbal fluency, semantic |
| Verbal Fluency, letter S (9) | Number of correct words generated in 60 sec | Verbal fluency, phonemic |
| WMS-III Spatial Span total (10) | Sum of raw scores on forward and backward conditions | Visual working memory |
| WMS-III Letter Number Sequencing ^2^ (10) | Total score | Verbal working memory |

^1^ Transformed 1/time, higher values indicate better performance also in these tasks (x1000)

^2^ A subsample (n=22) of the participants in Turku had been tested with the Matrics Letter–Number Span, and this data was transformed to equate the WMS Letter Number Sequencing data.

Supplementary Table 2. Standardized factor loadings and intercepts.

| Task | Standardized factor loadings | | Intercepts |
| --- | --- | --- | --- |
|  | Verbal factor | Visuomotor factor |  |
| WMS-III Letter Number Sequencing | 0.60 |  | 10.620 |
| Verbal Fluency, animals | 0.76 |  | 24.636 |
| Verbal Fluency, letter S | 0.65 |  | 15.427 |
| WAIS-III Vocabulary | 0.60 |  | 46.775 |
| Trail Making A, Inverted (× 1000) |  | 0.65 | 38.936 |
| Trail Making B, Inverted (× 1000) |  | 0.74 | 18.393 |
| WMS-III spatial span total |  | 0.49 | 18.292 |
| WAIS-III Digit Symbol |  | 0.82 | 75.422 |

Quantification of circulating cyto- and chemokines

Samples were allocated on the plates so that samples from the HEPS and TEPS were balanced on each plate, and samples from both time points from each individual were analyzed using the same plate. Luminex-analyses were performed with single reactions. Quantification of the markers was performed with Bio-plex 200 Luminex-instrument and Bio-Plex Manager software (Bio-Rad, Sweden). Concentration of each marker was determined from an 8-point standard curve using five parameter logistic regression. Minimum detectable concentration (MinDC) was determined for each marker separately using the lowest concentration on the standard curves linear phase (MinDC=c(low)+2SD). The samples below MinDC were given a value of 50% of MinDC. CCL17 levels were analysed in duplicate reactions and with 1:2 dilution of the serum samples with a commercial Human CCL17 (TARC) ELISA kit according to the manufactures instructions. 100µl of the standards and samples were diluted 1:2 in Assay Diluent C and were pipetted into the wells of the assay plate and incubated overnight at +4^o^C. The plate was then washed four times with the washing buffer provided with the kit and 100µl of streptavidin-HRP Reagent was added to each well. The plate was incubated at room temperature for 45 minutes followed by washing four times with the washing buffer. Then 100µl of TMB substrate was added to each well and the plate was allowed to develop at room temperature in the dark for 30 minutes. After the development phase 50µl of Stop Solution was added in the wells and the absorbance was measured using Multiskan Ascent plate reader (Thermo Fisher Scientific). The concentration of each sample was calculated using a standard curve and a four-parameter logistic regression.

Positron emission tomography

The radiotracer [^11^C]PBR28 was synthesized as described previously (11). Radiochemical purities were better than 99.9% and mean and standard deviation of molar activity at the end of synthesis were 506 and 147 MBq/nmol respectively. Foam padding and a restraining head strap was used to minimize head movement during the scan. Emission data were reconstructed using a 3D-OSEM algorithm into 23 frames of increasing length (6x0.5 min, 3x1 min, 2x2 min and 12x5 min) with a 2.73x2.73x3.27 mm^3^ voxel-size (12). Head motion during the scan was corrected by realigning all PET frames to the 12th frame containing the highest uptake on average. Individual frame-to-frame motion correction parameters were inspected and motion exceeding 5mm in any direction was used as a cutoff for exclusion.

Arterial blood sampling and radiometabolite analyses for PET

An automatic sampling system (Allogg ABSS, Mariefred, Sweden) was used to measure arterial whole blood radioactivity for the first 3 minutes of each scan. After that, arterial blood samples were manually drawn at 4, 6, 8, 10, 15, 20, 25, 30, 40, 50 and 70 min post injection of [^11^C]PBR28. Plasma and blood cells were immediately separated by centrifugation for 5 min at +4 °C (~2100 g) and their radioactivity was measured in a gamma counter (1480 Wizard^TM^ 3”, Wallac Oy, Turku, Finland)).

High-performance liquid chromatography (HPLC) was used to determine the fraction of parent [^11^C]PBR28 radioactivity in arterial plasma. Arterial blood samples at 4, 6, 10, 20, 30, 40 and 50 min post injection were manually drawn for the analysis of radiometabolites and unchanged [^11^C]PBR28 in plasma during the course of the PET scan. Each blood sample was immediately centrifuged for 5 min at +4 °C (~2100 g) to separate the plasma from the blood cells. Plasma samples (500 µl) were then mixed with acetonitrile (700 µl) to precipitate plasma proteins, and the plasma/acetonitrile mixture was centrifuged for 3 min at 3,400 g. 1 ml of the supernatant was then injected into a radio-HPLC system consisting of an interface module (LaChrom® Interface D-7000; Hitachi, Tokyo, Japan), a pump (L-7100 pump, Hitachi, Tokyo, Japan), an injector with a 1 ml loop (Rheodyne, Cotati, CA, USA) equipped with a column (Luna 5 µm C18 (2), 100 Å, 10 x 250 mm, Phenomenex, Torrance, USA) and an ultraviolet absorption detector (L-7400, 254 nm; Hitachi, Tokyo, Japan) in series with a lead-shielded radiation detector (Radiomatic 150TR, Perkin Elmer, Inc., USA). Acetonitrile (A) and ammonium formate (0.1 M HCOONH4) (B) were used as the mobile phase at 5.0 ml/min, according to the following gradient: 0 – 6 min A:B 40:60 v/v →  80:20 v/v; 6 – 8 min A:B 80:20 v/v; 8.1 – 10 min A:B 80:20 v/v  →  40:60 v/v. Chromatograms with peaks for radioactive compounds were integrated and the peak areas were expressed as a percentage of the sum of the areas of all detected radioactive compounds (decay-corrected to the time of injection into the HPLC system).

PET plasma input curve preprocessing

Decay corrected whole blood tissue activity curves (TAC) derived from automatic blood pump sampling were converted to plasma activity using hematocrit and a population derived distribution function specific for the tracer [^11^C]PBR28 and TSPO binding genotype. Automated and manual plasma sample TACs were then combined. The measured and estimated plasma activities were corrected for the fraction of unchanged tracer, which was interpolated using a Hill-function fit to the measured un-metabolized fraction time series. The time delay of the peak radioactivity reaching tissue and blood samples were corrected using PET count rate curves to reference peak tissue activity. Linear interpolation between the closest two values was used to approximate plasma activity during PET frames without blood sampling. The resulting plasma activity concentration curve, corrected for metabolites, was used as parent input for modeling. One HAB subject was missing automated blood samples for the first 0-5 minutes. For this subject a population based input function (PBIF) curve was utilized for the missing part. The PBIF curve was created using control subject HAB data imaged previously at Turku PET centre (n=34) (11). Each metabolite corrected plasma TAC was first standardized by the injected activity and the subject's body surface area using the Mosteller formula (13). Each standardized curve was then interpolated to have the same time points and shifted to the median peak time, after which an average PBIF curve for 0-4 min was calculated. The PBIF curve was then scaled to match the first manual sample at 4 min, whereafter the scaled PBIF curve was combined with the metabolite corrected manual samples to form a plasma input curve. Finally, this plasma curve was corrected for delay between tissue and input.

Tests for group effects in blood data

To assess sources of variation in V_T,_ statistical tests of blood data were done with group status as the independent variable. Areas under curve (AUC) for parent input and unchanged tracer fraction time series were calculated with the linear trapezoidal method using GraphPad Prism version 8.00 (GraphPad Software, La Jolla California USA, www.graphpad.com). Group differences of whole parent input AUC means were compared with Student’s t-test. Repeated measures analysis of variance (rANOVA) within the general linear model framework (GLM) was used to test for a significant group effect or time*sex interaction in unchanged tracer fractions.

PET tissue data characterization

Individual tissue time activity curves (TTAC) were visually inspected for quality. There were no significant hemispheric differences of regional VT (df=1, F=1.951, p=0.175) in an rANOVA model including study group and binding status as between subjects factors, with all 17 ROIs and two hemispheres as within-subject factor levels. VT from VOIs containing both hemispheres from the amygdala, anterior cingulate cortex, cerebellum, frontal cortex, hippocampus, insula, nucleus caudatus, occipital cortex, orbitofrontal cortex, parahippocampal cortex, parietal cortex, posterior cingulate cortex, prefrontal cortex, pallidum, putamen, temporal cortex and thalamus were chosen for further statistical testing.

PET statistics

For the PET study Shapiro-Wilks test and Q-Q plots were used to assess the normality of continuous variable distributions in each study group separately. The normality of V_T_ data was assessed using residuals of a rANOVA model with TSPO binding genotype as an independent variable. Correlations were assessed with Pearson’s r or Spearman’s rho correlations depending on variable distribution.

Voxel-wise modeling of [^11^C]PBR28 and statistical testing of V_T_

Parametric images of V_T_ were calculated as follows. First, the skull signal was masked out to minimize the effect of Gaussian filtering extracortical [^11^C] activity onto the cortical signal. The motion corrected PET images were then Gaussian filtered (FWHM 3 mm) to increase signal to noise ratio. Freely available in-house software was used to calculate voxel-wise V_T_  (<http://www.turkupetcentre.net/software/>) using Logan plot. Modeling was started from the frame starting at 30 minutes without V_T_ constraints. The parametric volume was then Gaussian filtered (FWHM 6 mm) and normalized to standard MNI space using transformations obtained by aligning the T1 weighted MR image to a standard MNI template using SPM12. An independent sample t-test was done with SPM12 to compare groups at voxel level using TSPO binding genotype as a covariate. The threshold of statistical significance used in the VOI level tests of group difference (p<0.05) was used as peak threshold for corresponding parametric t-tests. In all parametric analyses the family wiser error rate (FEW) corrected cluster threshold was p_FWE_<0.05 and the extent threshold was adjusted to the size of the smallest significant cluster.

References

1. Ventura J, Lukoff D, Nuechterlein K, Liberman RP, Green MF, Shaner A (1993): *Brief Psychiatric Rating Scale Expanded version 4.0: Scales anchor points and administration manual*. *Int J Meth Psychiatr Res*. (Vol. 13).

2. First M, Spitzer R, Gibbon M, Williams J (2002): *Structured Clinical Interview for DSM-IV-TR Axis I Disorders, Research Version, Non-patient Edition. (SCID-I/NP)*. New York, NY: Biometrics research, New York State Psychiatric Institute.

3. McGlashan T, Walsh B, Woods S (2010): *The Psychosis-Risk Syndrome: Handbook for Diagnosis and Follow-up*. New, York, NY: Oxford University Press.

4. Suvisaari J, Aalto-Setälä T, Tuulio-Henriksson A, Härkänen T, Saarni SI, Perälä J, *et al.* (2009): Mental disorders in young adulthood. *Psychol Med*. 39: 287–299.

5. Kalk NJ, Owen DR, Tyacke RJ, Reynolds R, Rabiner EA, Lingford-hughes AR, Parker CA (2013): Are prescribed benzodiazepines likely to affect the availability of the 18 kDa translocator protein (TSPO) in PET studies? *Synapse*. 67: 909–912.

6. Muthén LK, Muthén BO (2007): *Mplus User’s Guide. 5th*. (Vol. 7).

7. Reitan RM, Wolfson D (1993): *Halstead-Reitan Neuropsychological Test Battery: Theory and Clinical Interpretation*, 2nd ed. Tucson, Arizona: Neuropsychology Press.

8. Weschler D (1997): *Wechsler Adult Intelligence Scale*, 3rd ed. San Antonio, Texas: The Psychological Corporation.

9. Benton AL (1976): *Multilingual Aphasia Examination*, 1st ed. Iowa City, Iowa: University of Iowa.

10. Weschler D (1997): *Weschler Memory Scale*, 3rd ed. San Antonio, Texas: The Psychological Corporation.

11. Tuisku J, Plavén-Sigray P, Gaiser EC, Airas L, Al-Abdulrasul H, Brück A, *et al.* (2019): Effects of age, BMI and sex on the glial cell marker TSPO — a multicentre [11C]PBR28 HRRT PET study. *Eur J Nucl Med Mol Imaging*. 46: 2329–2338.

12. Johansson J, Teuho J, Lindén J, Tuna U, Tolvanen T, Saunavaara V, Teräs M (2013): Image quantification in high-resolution PET assessed with a new anthropomorphic brain phantom. *2013 IEEE Nucl Sci Symp Med Imaging Conf (2013 NSS/MIC)*. IEEE, pp 1–7.

13. Mosteller R (1987): Simplified Calculation of Body-Surface Area. *N Engl J Med*. 317: 1098–1098.
