# Supplementary material for: Elevated serum chemokine CCL22 levels in first-episode psychosis: associations with symptoms, peripheral immune state and in vivo brain glial cell function
Source: Transl Psychiatry. 2020 Mar 16;10:94. doi: 10.1038/s41398-020-0776-z (PMC7075957; doi:10.1038/s41398-020-0776-z)
Supplement: Supplementary file 2 — Supplementary Results File [file 41398_2020_776_MOESM2_ESM.docx]

# Supplementary Results File

## Descriptives and clinical characteristics

More FEP patients than controls were recruited in Helsinki, while the reverse was true in Turku. Median sample storage time was longer in Helsinki than in Turku. In both sites, controls had more years of education than patients but did not differ from patients in age or BMI. There were more antipsychotic-naïve patients in Turku than in Helsinki, and the median chlorpromazine equivalent dose was consequently higher in Helsinki than in Turku.

## PET descriptives and clinical characteristics

For the PET study FEPs and HCs did not significantly differ in years of education (*t*(26)=0.906, *p*=0.373), BMI (*U*=85.0, *p*=0.586), AUDIT-score (t(23)=-1.909, p=0.069), cannabis use (lifetime, χ^2^=1.418, p=0.395; past year, χ^2^=0.824, p=0.556). See Supplementary Figures 1 and 2 for group-wise time-series of peak and tail portions of metabolite corrected plasma input. There were no group differences in average movement during PET scan (t(26)=-0.567, p=0.575), injected tracer activity (U=92.5, p=0.821), unmetabolized tracer fractions (F(1,26)=0.512, p=0.481; Supplementary Figure 3) or cortical grey matter (GM) volume (t(26)=-0.292, p=0.773). Further, there were no significant differences of region of interest volume between FEP patients and controls (F(1,26)=0.405, p=0.530), or interaction between group status and region volume (F(1.931, 50.218)=0.581, p=0.558) in a repeated measures analysis of variance model. FEPs were significantly younger than HCs (t(26)=2.146, p=0.041), and more FEPs were tobacco smokers (χ^2^=5.791, p=0.029). See Supplementary Table 3 for details. There were no significant differences of TSPO genotype adjusted total GM V_T_ by sex (HC, t(13)=-1.455, p=0.169; FEP, t(11)=-0.169 , p=0.869) or correlations with age (HC, *ρ*=0.174, *p=*0.536; FEP, *ρ*=-0.334, *p*=0.264). BMI was significantly associated to TSPO genotype adjusted total GM V_T_ in HCs (*ρ*=-0.579, p=0.024), but not in FEPs (*ρ*=-0.516, p=0.071). See Supplementary Table 5 for individual clinical and imaging information of FEP group subjects. There were no significant associations to DUP (F(1,11)=0.337, p=0.573), total BPRS score (F(1,11)=0.260, p=0.621) or BPRS positive symptom score (F(1,11)=0.063, p=0.806) in FEPs.

## Associations of VT to peripheral CCL17 and CCL22 levels

For CCL17 the best fit linear regression lines of the FEP (F(1,11)=0.428, p=0.527) and HC (F(1,13)=2.808, p=0.118) groups were not significant. For CCL22 the best fit linear regression lines of the FEP (F(1,11)=1.977, p=0.187) and HC (F(1,13)=0.795, p=0.389) groups separately were not significant.

Supplementary Table 3. Characteristics of patients and controls recruited from the two study sites. Patient diagnoses according to DSM-IV criteria based on one-year follow-up.

|  | Helsinki | | | Turku | | |
| --- | --- | --- | --- | --- | --- | --- |
| Variable | **Patients  (n= 82)** | **Controls**  **(n=49)** | ***p* value^1^** | **Patients (n=47)** | **Controls**  **(n=81)** | ***p* value^1^** |
| Age, Years, Md (Q1, Q3) | 24.9  (22.0, 30.0) | 24.1  (22.4, 28.7) | 0.95 | 25.0  (22.0, 31.0) | 26.0  (23.0, 32.0) | 0.26 |
| Male/Female, n | 56/26 | 31/18 | 0.56 | 26/21 | 28/53 | **0.022** |
| BMI,  Md (Q1, Q3) | 22.8  (21.1, 25.7) | 23.9  (21.9, 25.8) | 0.29 | 23.6  (21.7, 29.1) | 24.1  (21.7, 27.3) | 0.95 |
| Years of education, Md (Q1, Q3) | 12.5  (12.0, 16.0) | 15.0  (13.0, 16.5) | **0.006** | 13.0  (12, 14.5) | 15.5  (14, 17) | **<.0001** |
| Sample storage time, days,  Md (Q1, Q3) | 1571  (1251, 2204) | 1483  (850, 1951) | **0.031** | 836  (429, 1019) | 417  (320, 845) | 0.050 |
| Chlorpromazine equivalent dose | 300  (180, 450) | - | **-** | 167  (0, 300) | - | **<.0001** |
| Schizophrenia (n) | 30 | - | **-** | 12 | - | **-** |
| Schizophreniform disorder (n) | 21 | - | **-** | 9 | - | **-** |
| Psychosis NOS (n) | 14 | - | **-** | 11 | - | **-** |
| Bipolar I disorder with psychotic features (n) | 7 | - | **-** | 3 | - | **-** |
| MDD with psychotic features (n) | 4 | - | **-** | 4 | - | **-** |
| Delusional disorder (n) | 1 | - | **-** | 5 | - | **-** |
| Schizoaffective disorder (n) | 2 | - | **-** | 2 | - | **-** |
| Brief psychotic disorder (n) | 3 | - | **-** | 1 | - | **-** |

NOS=Not otherwise specified, MDD=major depressive disorder, Md=median, Q1=first quartile, Q3=third quartile

^1^ χ^2^ test used for dichotomous variables, Wilcoxon two-sample test with *t* approximation used for ordinal and continuous variables

Supplementary Table 4. Demographic, clinical and imaging information of PET study groups

|  | FEP | HC | *p* value |
| --- | --- | --- | --- |
| Number of subjects (n) | 13 | 15 |  |
| Male sex (n) | 7 | 5 | 0.45 |
| TSPO binding genotype (n)* | 8/5 | 9/6 | 0.62 |
| Age (years) | 24.8±4 | 29.7±7 | <0.05 |
| BMI (kg m^-2^) | 23.5 | 24.0 | 0.59 |
| Years of education | 15±3 | 16±3 | 0.37 |
| GAF | 42±18 | 90±4 | <0.05 |
| BPRS-E score | 60±18 | 28±1 | <0.05 |
| Tobacco smokers (n) | 6 | 1 | <0.05 |
| AUDIT score | 11±7** | 7±3 | 0.10 |
| Lifetime cannabis use >5 times (n) | 9** | 9 | 0.40 |
| Past year cannabis use >5 times (n) | 3** | 0 | 0.56 |
| Injected [^11^C]PBR28 activity (MBq) | 400.2±15 | 400.1±18 | 0.98 |
| Injected [^11^C]PBR28 mass (µg) | 0.61±0.13 | 0.55±0.18 | 0.35 |
| Un-metabolized tracer fraction AUC (1*min) | 1491±283 | 1410±330 | 0.48 |
| Average frame-to-frame movement (mm) | 0.44±0.16 | 0.42±0.10 | 0.58 |

Values are number, median, or mean ± standard deviation. Abbreviations: AUC, area under curve; AUDIT, Alcohol Use Disorders Identification Test; BMI, body mass index; BPRS‑E, Brief Psychiatric Rating Scale – Extended; FEP, first-episode psychosis; GAF, Global Assessment of Functioning; HC, healthy controls. *: high-affinity binder/medium-affinity binder; **: n=11

Supplementary Table 5. Individual clinical and imaging information of first episode psychosis PET subjects

| Parti­cipant | Age (years) | Sex | Smoking | GAF | DSM-IV-TR diagnosis | BPRS-E score sum | Benzodiazepine medication/d | Antipsychotic medication/d | DOI (days) | TSPO genotype | Total GM V_T_ |
| --- | --- | --- | --- | --- | --- | --- | --- | --- | --- | --- | --- |
| #1 | 26 | Female | No | 60 | 298.9x | 27 | - | Olanzapine 20mg | 228 | HAB | 3.72 |
| #2 | 30 | Male | Yes | 60 | 298.9x | 35 | - | Olanzapine 10mg | 99 | HAB | 4.22 |
| #3 | 27 | Female | No | 30 | 295.30 | 44 | - | Risperidone 2mg | 178 | MAB | 1.93 |
| #4 | 26 | Female | Yes | 30 | 295.40 | 55 | Lorazepam 2mg | Olanzapine 15mg | 33 | MAB | 2.97 |
| #5 | 19 | Male | No | 61 | 296.24 | 46 | - | Olanzapine 7.5mg | 67 | MAB | 2.94 |
| #6 | 24 | Male | Yes | 30 | 295.70 | 64 | - | Quetiapine 325mg,  Aripiprazole 15mg | 21 | HAB | 5.40 |
| #7 | 28 | Female | Yes | 55 | 295.70 | 37 | Lorazepam 1mg | - | 336 | HAB | 4.39 |
| #8 | 20 | Male | Yes | 40 | 298.9x | 45 | - | Olanzapine 7.5mg | 22 | HAB | 5.70 |
| #9 | 30 | Male | No | 30 | 295.40 | 72 | - | Risperidone 2mg,  Olanzapine 10mg | 37 | HAB | 4.42 |
| #10 | 22 | Female | No | 40 | 298.8x | 62 | Oxazepam 15mg | Aripiprazole 15mg | 99 | MAB | 3.99 |
| #11 | 20 | Female | No | 10 | 296.24 | 68 | - | Olanzapine 10mg | 101 | MAB | 1.86 |
| #12 | 21 | Male | Yes | 31 | 295.70 | 71 | - | Paliperidone 2.5mg,  Quetiapine 25mg | 269 | HAB | 3.17 |
| #13 | 29 | Male | No | 70 | 296.04 | 36 | - | - | 55 | HAB | 3.94 |
| #14 | 37 | Male | No | 55 | 296.04 | 47 | - | Quetiapine 650mg | 36 | LAB | 0.67 |

Abbreviations: BPRS-E, Brief Psychiatric Rating Scale - Extended; DSM-IV-TR, Diagnostic and Statistical Manual for Mental Disorders, 4^th^ Edition, text Revision; DOI, duration of illness; GAF, Global Assessment of Functioning; GM, grey matter; HAB, high-affinity binding genotype; LAB, low-affinity binding genotype; MAB, medium-affinity binding genotype.

Supplementary Table 6. Association of serum CCL22 levels with potential confounding variables

| Variable | CCL22 level (pg/mL)  Md (Q1, Q3) | *p* value (Wilcoxon two-sample test) |
| --- | --- | --- |
| Sex: |  |  |
| Men (n=141) | 1114.4 (847.9, 1436.8) | 0.15 |
| Women (n=118) | 1030.2 (818.6, 1404.4) |  |
| Current smoking: |  |  |
| Yes (n=39) | 1325.1 (1014.6, 1867.1) | **0.0005** |
| No (n=183) | 1022.4 (802.8, 1301.6) |  |
| Cannabis use past 12 months: |  |  |
| Yes (n=54) | 1093.9 (807.1, 1325.1) | 0.89 |
| No (n=183) | 1044.2 (825.5, 1354.9) |  |
| Obesity (BMI ≥ 30): |  |  |
| Yes (n=27) | 1306.8 (961.0, 2280.6) | **0.006** |
| No (n=226) | 1048.3 (819.6, 1334.1) |  |
| Olanzapine use (in patients): |  |  |
| Yes (n=45) | 1340.1 (1113.8, 1887.8) | **0.030** |
| No (n=84) | 1159.6 (912.8, 1536.7) |  |
| Risperidone use (in patients): |  |  |
| Yes (n=45) | 1175.6 (1006.4, 1693.0) | 0.99 |
| No (n=84) | 1277.5 (1014.6, 1600.0) |  |
| Any antipsychotic (in patients): |  |  |
| Yes (n=113) | 1287.3 (1032.1, 1693.0) | 0.29 |
| No (n=16) | 1098.1 (846.7, 1494.0) |  |

Md=median, Q1=first quartile, Q3=third quartile

Supplementary Table 7. Correlation of serum CCL22 with other serum cytokine and chemokine levels in patients with first-episode psychosis and controls

|  | Correlation (*ρ*)^1^ with CCL22 in patients (N=129) | p value | Correlation (*ρ*)^1^ with CCL22 in controls (N=130) | *p* value |
| --- | --- | --- | --- | --- |
| CCL2 | 0.33 | **0.0001** | 0.21 | **0.02** |
| CCL3 | 0.26 | 0.003 | 0.34 | **<.0001** |
| CCL4 | 0.28 | 0.001 | 0.32 | 0.0002 |
| CCL7 | 0.10 | 0.25 | 0.19 | 0.03 |
| CCL11 | 0.09 | 0.32 | 0.18 | **0.04** |
| CX3CL1 | 0.15 | 0.09 | 0.31 | **0.0003** |
| CXCL1 | 0.04 | 0.66 | 0.02 | 0.85 |
| CXCL10 | 0.18 | 0.05 | 0.12 | 0.16 |
| EGF | 0.08 | 0.37 | 0.17 | 0.05 |
| FGF-2 | 0.19 | **0.03** | 0.31 | **0.0003** |
| FLT-3L | 0.24 | **0.006** | 0.41 | **<.0001** |
| G-CSF | 0.12 | 0.17 | 0.13 | 0.13 |
| GM-CSF | 0.28 | **0.001** | 0.28 | **0.001** |
| IFN-α2 | 0.05 | 0.59 | 0.29 | **0.0009** |
| IFN-γ | 0.19 | **0.03** | 0.38 | **<.0001** |
| IL-1α | 0.02 | 0.82 | 0.25 | **0.004** |
| IL-1β | 0.18 | **0.04** | 0.38 | **<.0001** |
| IL-1RA | 0.17 | 0.05 | 0.23 | **0.008** |
| IL-2 | 0.10 | 0.25 | 0.25 | **0.004** |
| IL-3 | -0.05 | 0.59 | 0.13 | 0.15 |
| IL-4 | 0.04 | 0.68 | 0.17 | 0.05 |
| IL-5 | 0.13 | 0.14 | 0.18 | 0.05 |
| IL-6 | 0.09 | 0.32 | 0.27 | **0.002** |
| IL-7 | 0.01 | 0.95 | 0.25 | **0.004** |
| IL-8 | 0.24 | **0.008** | 0.24 | **0.007** |
| IL-9 | 0.03 | 0.71 | 0.26 | **0.003** |
| IL-10 | 0.02 | 0.82 | 0.23 | **0.009** |
| IL12-p40 | -0.08 | 0.39 | 0.14 | 0.10 |
| IL12-p70 | 0.19 | **0.03** | 0.33 | **0.0001** |
| IL-13 | 0.07 | 0.44 | 0.19 | **0.03** |
| IL-15 | 0.07 | 0.42 | 0.22 | **0.01** |
| IL-17 | 0.23 | **0.009** | 0.31 | **0.0003** |
| sCD40L | 0.18 | **0.04** | 0.11 | 0.22 |
| TGF-α | 0.40 | **<.0001** | 0.23 | **0.008** |
| TNF-α | 0.29 | **0.0007** | 0.44 | **<.0001** |
| TNF-β | 0.06 | 0.49 | 0.10 | 0.24 |
| VEGF | 0.30 | **0.0006** | 0.37 | **<.0001** |

^1^ Spearman rank order correlation coefficient

Abbreviations: CCL2 = C-C motif chemokine 2 = Monocyte chemoattractant protein 1 (MCP-1); CCL3 = macrophage inflammatory protein 1-alpha (MIP-1α); CCL4 = macrophage inflammatory protein 1-beta (MIP-1β); CCL7 = monocyte-chemotactic protein 3 (MCP-3); CCL11 = eotaxin; CCL22 = macrophage derived chemokine; CX3CL1 = chemokine (C-X3-C motif) ligand = fractalkine; CXCL1 = chemokine (C-X-C motif) ligand 1 = GROα; CXCL10 = IP10 (Interferon gamma-induced protein 10); EGF = Epidermal growth factor; FGF-2 = basic fibroblast growth factor; FLT-3L = Fms-related tyrosine kinase 3 ligand; G-CSF = Granulocyte-colony stimulating factor; GM-CSF = human granulocyte-macrophage colony–stimulating factor; IFN = Interferon; IL= interleukin; IL-1RA = interleukin 1 receptor antagonist; IL-8 = chemokine (C-X-C motif) ligand 8; IL12-p40 = Subunit beta of interleukin 12 (common subunit for IL-12 and IL-23; IL12B); IL12-p70 = the active heterodimer of IL-12; sCD40L = soluble CD-40 ligand; TGF-α = Transforming growth factor alpha; TNF-α = tumor necrosis factor-alpha; TNF-β = tumor necrosis factor-beta; VEGF = vascular endothelial growth factor

Supplementary Table 8. Serum cytokine and chemokine levels in patients vs. controls with high CCL22

|  | High CCL22 patients (n=64)  Md (Q1, Q3) | High CCL22 controls (n=24)  Md (Q1, Q3) | *p* value^1^ |
| --- | --- | --- | --- |
| CCL2 | 726.4 (585.9,1016.5) | 671.2 (588.1, 826.8) | 0.20 |
| CCL3 | 28.2 (15.8, 46.3) | 34.5 (15.8, 60.0) | 0.20 |
| CCL4 | 86.5 (46.1, 158.8) | 148.5 (45.2, 263.4) | 0.11 |
| CCL7 | 44.4 (1.9, 279.7) | 115.5 (1.9, 314.4) | 0.47 |
| CCL11 | 166.8 (123.3, 223.3) | 144.6 (120.0, 224.5) | 0.60 |
| CCL22 | 1625.3 (1407.4, 2001.2) | 1647.3 (1404.4, 2075.7) | 0.64 |
| CX3CL1 | 66.1 (11.4, 175.0) | 247.0 (92.0, 493.7) | **0.0009** |
| CXCL1 | 990.3 (750.8,1284.3) | 1111.9 (870.9,1333.0) | 0.22 |
| CXCL10 | 248.6 (197.5, 295.6) | 245.5 (195.4, 343.3) | 0.33 |
| EGF | 132.5 (83.1, 229.5) | 171.0 (96.5, 232.5) | 0.33 |
| FGF-2 | 92.6 (40.2, 151.3) | 148.0 (84.2, 503.0) | **0.011** |
| FLT-3L | 2.7 (2.7, 24.2) | 27.2 (2.7, 141.7) | **0.008** |
| G-CSF | 45.1 (7.1, 87.2) | 69.8 (0.9, 130.9) | 0.44 |
| GM-CSF | 14.2 (3.8, 37.1) | 25.2 (12.2, 124.0) | **0.018** |
| IFN-α2 | 20.0 (1.5, 55.9) | 104.4 (1.5, 185.9) | **0.009** |
| IFN-γ | 24.6 (8.9, 81.1) | 75.1 (23.7, 242.6) | **0.010** |
| IL-1α | 23.6 (4.7, 147.7) | 102.8 (4.7, 261.5) | 0.07 |
| IL-1β | 2.1 (0.4, 4.4) | 4.4 (2.1, 11.8) | **0.007** |
| IL-1RA | 34.7 (4.2, 253.3) | 99.4 (4.2, 460.3) | 0.23 |
| IL-2 | 4.9 (0.5, 15.1) | 22.8 (0.5, 78.0) | **0.039** |
| IL-3 | 0.4 (0.4, 3.5) | 0.4 (0.4, 6.0) | 0.10 |
| IL-4 | 2.3 (2.3, 11.6) | 2.3 (2.3, 8.8) | 0.26 |
| IL-5 | 2.9 (0.3, 29.1) | 4.3 (0.3, 48.6) | 0.72 |
| IL-6 | 13.5 (0.5, 53.3) | 30.3 (0.5, 104.3) | 0.05 |
| IL-7 | 3.1 (0.7, 13.3) | 14.4 (1.4, 41.0) | **0.003** |
| IL-8 | 47.1 (19.6, 88.2) | 40.6 (18.1, 100.7) | 0.59 |
| IL-9 | 3.3 (0.6, 8.3) | 9.5 (0.6, 23.9) | 0.11 |
| IL-10 | 5.6 (0.6, 29.1) | 28.9 (0.6, 86.4) | 0.13 |
| IL12-p40 | 3.7 (3.7, 98.0) | 65.5 (3.7, 196.3) | 0.07 |
| IL12-p70 | 12.1 (0.3, 91.9) | 41.4 (16.6, 338.0) | **0.011** |
| IL-13 | 32.1 (0.7, 203.6) | 37.1 (0.7, 186.4) | 0.66 |
| IL-15 | 7.9 (0.6, 20.5) | 22.7 (0.6, 79.4) | 0.06 |
| IL-17 | 27.3 (5.0, 60.7) | 38.6 (26.0, 129.5) | **0.031** |
| sCD40L | 3403.5 (2426.8, 4279.2) | 3238.0 (2506.8, 3900.7) | 0.84 |
| TGF-α | 10.0 (6.6, 20.4) | 18.0 (6.1, 36.9) | 0.14 |
| TNF-α | 17.1 (12.8, 24.5) | 24.0 (11.9, 47.2) | 0.18 |
| TNF-β | 0.8 (0.8, 228.6) | 17.8 (0.8, 211.2) | 0.75 |
| VEGF | 424.7 (243.4, 705.1) | 469.9 (248.6,1027.3) | **0.41** |

^1^ Wilcoxon signed rank test

Supplementary Table 9. Baseline serum cytokine and chemokine levels (pg/mL) in patients and controls with high or low CCL22 defined according to the median CCL22 level in patients

|  | FEP patients | | | Controls | | |
| --- | --- | --- | --- | --- | --- | --- |
|  | **High CCL22 (n=64)**  **Md (Q1, Q3)** | **Low CCL22 (n=65)**  **Md (Q1, Q3)** | ***p* value^1^** | **High CCL22**  **(n=24)**  **Md (Q1, Q3)** | **Low CCL22**  **(n=106)**  **Md (Q1, Q3)** | ***p* value^1^** |
| CCL2 | 726.4  (585.9,1016.5) | 648.6  (515.0, 821.8) | **0.016** | 671.2  (588.1, 826.8) | 643.3  (521.1, 796.8) | 0.37 |
| CCL3 | 28.2  (15.8, 46.3) | 19.6  (9.5, 29.4) | **0.0015** | 34.5  (15.8, 60.0) | 18.4  (8.5, 33.1) | **0.0005** |
| CCL4 | 86.5  (46.1, 158.8) | 47.8  (26.1, 103.7) | **0.0013** | 148.5  (45.2, 263.4) | 58.2  (25.2, 109.7) | **0.0003** |
| CCL7 | 44.4  (1.9, 279.7) | 1.9  (1.9, 104.1) | 0.06 | 115.5  (1.9, 314.4) | 1.9  (1.9, 246.1) | 0.10 |
| CCL11 | 166.8  (123.3, 223.3) | 165.9  (117.4, 210.0) | 0.49 | 144.6  (120.0, 224.5) | 145.6  (117.5, 192.2) | 0.44 |
| CX3CL1 | 66.1  (11.4, 175.0) | 42.7  (11.4, 108.0) | 0.25 | 247.0  (92.0, 493.7) | 28.9  (11.4, 130.1) | **<.0001** |
| CXCL1 | 990.3  (750.8,1284.3) | 987.7  (727.8,1227.2) | 0.70 | 1111.9  (870.9,1333.0) | 1018.4  (843.0,1307.9) | 0.44 |
| CXCL10 | 248.6  (197.5, 295.6) | 230.9  (167.0, 297.4) | 0.33 | 245.5  (195.4, 343.3) | 211.6  (171.2, 262.6) | **0.014** |
| EGF | 132.5  (83.1, 229.5) | 114.3  (78.4, 206.7) | 0.26 | 171.0  (96.5, 232.5) | 125.7  (83.8, 194.4) | 0.06 |
| FGF-2 | 92.6  (40.2, 151.3) | 57.4  (27.9, 106.7) | **0.029** | 148.0  (84.2, 503.0) | 63.7  (40.2, 118.4) | **0.0001** |
| FLT-3L | 2.7  (2.7, 24.2) | 2.7  (2.7, 13.2) | **0.049** | 27.2  (2.7, 141.7) | 2.7  (2.7, 2.7) | **<.0001** |
| G-CSF | 45.1  (7.1, 87.2) | 28.9  (0.9, 72.0) | 0.09 | 69.8  (0.9, 130.9) | 11.2  (0.9, 81.5) | 0.06 |
| GM-CSF | 14.2  (3.8, 37.1) | 3.8  (3.8, 15.6) | **0.0019** | 25.2  (12.2, 124.0) | 3.8  (3.8, 15.9) | **<.0001** |
| IFN-α2 | 20.0  (1.5, 55.9) | 1.5  (1.5, 44.0) | 0.24 | 104.4  (1.5, 185.9) | 1.5  (1.5, 37.6) | **0.0013** |
| IFN-γ | 24.6  (8.9, 81.1) | 14.3  (5.2, 29.7) | **0.022** | 75.1  (23.7, 242.6) | 16.5  (7.1, 45.0) | **0.0002** |
| IL-1α | 23.6  (4.7, 147.7) | 24.8  (4.7, 106.7) | 0.58 | 102.8  (4.7, 261.5) | 9.7  (4.7, 111.8) | **0.007** |
| IL-1β | 2.1  (0.4, 4.4) | 0.4  (0.4, 2.4) | **0.024** | 4.4  (2.1, 11.8) | 0.4  (0.4, 1.7) | **<.0001** |
| IL-1RA | 34.7  (4.2, 253.3) | 4.2  (4.2, 70.6) | **0.017** | 99.4  (4.2, 460.3) | 4.2  (4.2, 193.7) | **0.012** |
| IL-2 | 4.9  (0.5, 15.1) | 1.4  (0.5, 14.1) | 0.15 | 22.8  (0.5, 78.0) | 0.5  (0.5, 13.4) | **0.0010** |
| IL-3 | 0.4  (0.4, 3.5) | 0.4  (0.4, 4.1) | 0.82 | 0.4  (0.4, 6.0) | 0.4  (0.4, 2.4) | **0.047** |
| IL-4 | 2.3  (2.3, 11.6) | 2.3  (2.3, 12.6) | 0.77 | 2.3  (2.3, 8.8) | 2.3  (2.3, 6.1) | 0.07 |
| IL-5 | 2.9  (0.3, 29.1) | 0.9  (0.3, 6.3) | **0.028** | 4.3  (0.3, 48.6) | 0.9  (0.3, 20.5) | 0.09 |
| IL-6 | 13.5  (0.5, 53.3) | 9.7  (0.5, 26.6) | 0.36 | 30.3  (0.5, 104.3) | 6.6  (0.5, 29.0) | **0.0016** |
| IL-7 | 3.1  (0.7, 13.3) | 3.0  (0.7, 11.0) | 0.71 | 14.4  (1.4, 41.0) | 0.7  (0.7, 10.5) | **0.0002** |
| IL-8 | 47.1  (19.6, 88.2) | 21.3  (11.4, 42.0) | **0.0010** | 40.6  (18.1, 100.7) | 27.1  (11.2, 55.1) | **0.025** |
| IL-9 | 3.3  (0.6, 8.3) | 2.8  (0.6, 9.7) | 0.67 | 9.5  (0.6, 23.9) | 0.6  (0.6, 7.0) | **0.010** |
| IL-10 | 5.6  (0.6, 29.1) | 5.2  (0.6, 29.2) | 0.70 | 28.9  (0.6, 86.4) | 0.6  (0.6, 20.5) | **0.007** |
| IL12-p40 | 3.7  (3.7, 98.0) | 3.7  (3.7, 119.1) | 0.66 | 65.5  (3.7, 196.3) | 3.7  (3.7, 78.8) | **0.040** |
| IL12-p70 | 12.1  (0.3, 91.9) | 2.8  (0.3, 32.1) | 0.07 | 41.4 (16.6, 338.0) | 5.0  (0.3, 27.4) | **<.0001** |
| IL-13 | 32.1  (0.7, 203.6) | 14.7  (0.7, 83.9) | 0.18 | 37.1  (0.7, 186.4) | 19.0  (0.7, 152.5) | 0.26 |
| IL-15 | 7.9  (0.6, 20.5) | 1.9  (0.6, 17.6) | 0.16 | 22.7  (0.6, 79.4) | 0.6  (0.6, 14.2) | **0.0012** |
| IL-17 | 27.3  (5.0, 60.7) | 10.1  (3.7, 27.2) | **0.0083** | 38.6  (26.0, 129.5) | 14.1  (6.1, 35.1) | **0.0002** |
| sCD40L | 3403.5 (2426.8, 4279.2) | 2694.2 (1864.8, 3759.7) | 0.07 | 3238.0 (2506.8, 3900.7) | 2954.1 (2108.9, 4050.0) | 0.27 |
| TGF-α | 10.0  (6.6, 20.4) | 5.0  (2.9, 8.9) | **<.0001** | 18.0 (6.1, 36.9) | 6.3 (4.1, 13.0) | **0.0020** |
| TNF-α | 17.1  (12.8, 24.5) | 12.7  (9.6, 17.5) | **0.0006** | 24.0  (11.9, 47.2) | 11.6  (9.8, 15.5) | **0.0001** |
| TNF-β | 0.8  (0.8, 228.6) | 0.8  (0.8, 65.9) | 0.14 | 17.8  (0.8, 211.2) | 0.8  (0.8, 205.0) | 0.43 |
| VEGF | 424.7  (243.4, 705.1) | 229.3  (148.1, 436.1) | **0.0006** | 469.9  (248.6,1027.3) | 249.8  (118.2, 428.2) | **0.0006** |

^1^ Wilcoxon two-sample test

Supplementary Table 10. One-year follow-up results on serum cytokine and chemokine levels in patients with first-episode psychosis and controls

|  | Patients (N=58)  Md (Q1, Q3) | Controls (N=55) Md (Q1, Q3) | Wilcoxon two-sample test (t approximation) Statistical significance *p* |
| --- | --- | --- | --- |
| CCL2 | 678.0 (549.9, 865.7) | 668.3 (536.7, 825.1) | 0.58 |
| CCL3 | 20.1 (10.5, 37.0) | 22.4 (12.0, 39.2) | 0.81 |
| CCL4 | 64.6 (27.4, 125.9) | 77.2 (30.8, 138.2) | 0.84 |
| CCL7 | 20.6 (1.9, 280.7) | 86.7 (1.9, 270.3) | 0.67 |
| CCL11 | 174.0 (132.9, 225.0) | 154.4 (133.6, 219.4) | 0.46 |
| CCL22 | 1097.7 (933.6, 1372.1) | 843.7 (745.4, 1126.0) | **0.0007** |
| CX3CL1 | 33.4 (11.4, 121.6) | 63.5 (11.4, 166.3) | **0.038** |
| CXCL1 | 1055.9 (810.9, 1345.8) | 942.9 (745.2, 1216.0) | 0.18 |
| CXCL10 | 205.6 (170.8, 315.6) | 237.9 (181.8, 324.0) | 0.51 |
| EGF | 113.8 (77.2, 221.4) | 148.9 (104.7, 226.3) | 0.35 |
| FGF-2 | 70.6 (41.0, 124.7) | 79.0 (48.0, 150.1) | 0.40 |
| FLT-3L | 2.7 (2.7, 19.2) | 2.7 (2.7, 26.5) | 0.74 |
| G-CSF | 40.9 (0.9, 94.0) | 40.9 (0.9, 94.4) | 0.98 |
| GM-CSF | 3.8 (3.8, 21.4) | 7.9 (3.8, 35.7) | 0.24 |
| IFN-α2 | 1.5 (1.5, 31.3) | 17.2 (1.5, 69.7) | **0.033** |
| IFN-γ | 17.0 (5.0, 46.1) | 28.9 (10.3, 61.8) | 0.26 |
| IL-1α | 47.2 (4.7, 156.9) | 43.2 (4.7, 192.6) | 0.87 |
| IL-1β | 0.4 (0.4, 2.4) | 1.5 (0.4, 4.0) | 0.24 |
| IL-1RA | 4.2 (4.2, 223.0) | 31.6 (4.2, 278.9) | 0.61 |
| IL-2 | 4.3 (0.5, 17.7) | 4.0 (0.5, 26.6) | 0.67 |
| IL-3 | 0.4 (0.4, 3.7) | 0.4 (0.4, 7.4) | 0.40 |
| IL-4 | 2.3 (2.3, 12.0) | 2.3 (2.3, 15.5) | 0.96 |
| IL-5 | 1.4 (0.3, 21.3) | 3.2 (0.3, 28.0) | 0.91 |
| IL-6 | 12.3 (0.5, 36.0) | 16.2 (0.5, 49.0) | 0.73 |
| IL-7 | 2.7 (0.7, 10.8) | 4.6 (0.7, 19.4) | 0.35 |
| IL-8 | 28.0 (13.0, 77.8) | 31.8 (15.8, 62.8) | 0.51 |
| IL-9 | 2.0 (0.6, 9.5) | 2.7 (0.6, 12.5) | 0.76 |
| IL-10 | 4.1 (0.6, 37.6) | 6.3 (0.6, 57.0) | 0.60 |
| IL12-p40 | 3.7 (3.7, 122.2) | 15.2 (3.7, 163.8) | 0.37 |
| IL12-p70 | 3.5 (0.3, 43.8) | 10.2 (0.3, 31.3) | 0.72 |
| IL-13 | 21.5 (0.7, 188.2) | 44.4 (0.7, 209.3) | 0.93 |
| IL-15 | 6.0 (0.6, 17.3) | 3.0 (0.6, 27.7) | 0.95 |
| IL-17 | 12.2 (4.5, 43.2) | 23.6 (5.8, 41.7) | 0.35 |
| sCD40L | 2689.0 (1946.2, 4192.2) | 2440.8 (1794.4, 3681.0) | 0.56 |
| TGF-α | 7.0 (3.6, 12.7) | 5.9 (3.2, 10.6) | 0.36 |
| TNF-α | 13.6 (10.9, 22.5) | 13.7 (10.0, 18.6) | 0.64 |
| TNF-β | 0.8 (0.8, 204.5) | 0.8 (0.8, 223.5) | 0.51 |
| VEGF | 349.0 (128.5, 488.0) | 269.7 (121.8, 529.3) | 0.49 |

Abbreviations: Md = median, Q1 = first quartile, Q3 = third quartile

CCL2 = C-C motif chemokine 2 = Monocyte chemoattractant protein 1 (MCP-1); CCL3 = macrophage inflammatory protein 1-alpha (MIP-1α); CCL4 = macrophage inflammatory protein 1-beta (MIP-1β); CCL7 = monocyte-chemotactic protein 3 (MCP-3); CCL11 = eotaxin; CCL22 = macrophage derived chemokine; CX3CL1 = chemokine (C-X3-C motif) ligand = fractalcine; CXCL1 = chemokine (C-X-C motif) ligand 1 = GROα; CXCL10 = IP10 (Interferon gamma-induced protein 10); EGF = Epidermal growth factor; FGF-2 = basic fibroblast growth factor; FLT-3L = Fms-related tyrosine kinase 3 ligand; G-CFS = Granulocyte-colony stimulating factor; GM-CSF = human granulocyte-macrophage colony–stimulating factor; IFN = Interferon; IL= interleukin; IL-1RA = interleukin 1 receptor antagonist; IL-8 = chemokine (C-X-C motif) ligand 8; IL12-p40 = Subunit beta of interleukin 12 (common subunit for IL-12 and IL-23; IL12B); IL12-p70 = the active heterodimer of IL-12; sCD40L = soluble CD-40 ligand; TGF-α = Transforming growth factor alpha; TNF-α = tumor necrosis factor-alpha; TNF-β = tumor necrosis factor-beta; VEGF = vascular endothelial growth factor

Supplementary Table 11. One-year follow-up cytokine and chemokine levels in patients with high and low baseline CCL22

|  | High baseline CCL22 (n=22)  Md (Q1, Q3) | Low baseline CCL22 (n=36)  Md (Q1, Q3) | *p* value^1^ |
| --- | --- | --- | --- |
| CCL2 | 678.9 (538.6, 934.1) | 662.9 (565.1, 839.5) | 0.77 |
| CCL3 | 24.8 (11.5, 42.9) | 19.6 (10.1, 27.8) | 0.23 |
| CCL4 | 105.8 (36.0, 191.4) | 60.4 (21.3, 101.0) | **0.042** |
| CCL7 | 20.9 (1.9, 388.6) | 1.9 (1.9, 130.7) | 0.32 |
| CCL11 | 186.4 (135.3, 246.2) | 160.0 (126.1, 219.2) | 0.43 |
| CCL22 | 1309.6 (1097.7, 1655.2) | 979.6 (872.4, 1176.0) | **0.0005** |
| CX3CL1 | 11.4 (11.4, 143.1) | 33.7 (11.4, 99.6) | 1.00 |
| CXCL1 | 1110.7 (911.0, 1318.8) | 869.9 (666.7, 1345.8) | 0.11 |
| CXCL10 | 214.5 (189.9, 275.9) | 199.8 (166.5, 315.6) | 0.87 |
| EGF | 142.5 (78.0, 241.3) | 110.0 (71.9, 217.3) | 0.73 |
| FGF-2 | 79.8 (65.8, 181.3) | 55.3 (35.8, 95.5) | **0.042** |
| FLT-3L | 2.7 (2.7, 34.3) | 2.7 (2.7, 15.9) | 0.63 |
| G-CSF | 36.8 (4.2, 107.0) | 40.9 (0.9, 86.1) | 0.72 |
| GM-CSF | 11.2 (3.8, 43.0) | 3.8 (3.8, 9.0) | **0.025** |
| IFN-α2 | 1.5 (1.5, 52.9) | 1.5 (1.5, 15.8) | 0.81 |
| IFN-γ | 36.1 (8.5, 69.6) | 10.0 (4.6, 36.2) | 0.14 |
| IL-1α | 30.7 (4.7, 252.4) | 47.2 (4.7, 109.4) | 0.66 |
| IL-1β | 1.6 (0.4, 3.5) | 0.4 (0.4, 2.1) | 0.10 |
| IL-1RA | 11.8 (4.2, 564.6) | 4.2 (4.2, 68.2) | 0.23 |
| IL-2 | 3.8 (0.5, 12.1) | 4.3 (0.5, 18.1) | 1.00 |
| IL-3 | 0.4 (0.4, 1.8) | 0.4 (0.4, 4.4) | 0.33 |
| IL-4 | 5.6 (2.3, 18.7) | 2.3 (2.3, 5.3) | **0.040** |
| IL-5 | 2.0 (0.3, 36.3) | 0.6 (0.3, 5.6) | 0.22 |
| IL-6 | 15.6 (0.5, 70.9) | 12.0 (0.5, 25.7) | 0.56 |
| IL-7 | 2.4 (0.7, 10.6) | 2.7 (0.7, 10.8) | 0.97 |
| IL-8 | 38.4 (21.3, 102.6) | 17.2 (11.3, 46.6) | **0.021** |
| IL-9 | 2.4 (0.6, 9.1) | 1.6 (0.6, 9.5) | 0.73 |
| IL-10 | 0.6 (0.6, 19.0) | 4.1 (0.6, 41.9) | 0.45 |
| IL12-p40 | 3.7 (3.7, 105.0) | 3.7 (3.7, 122.2) | 0.59 |
| IL12-p70 | 9.8 (0.3, 73.8) | 2.5 (0.3, 19.5) | 0.16 |
| IL-13 | 34.6 (0.7, 327.6) | 19.4 (0.7, 107.6) | 0.59 |
| IL-15 | 6.2 (0.6, 16.8) | 3.0 (0.6, 24.3) | 0.57 |
| IL-17 | 25.6 (5.7, 51.9) | 8.4 (3.9, 32.8) | 0.10 |
| sCD40L | 2756.0 (1946.2, 4451.6) | 2656.9 (1895.9, 3470.7) | 0.82 |
| TGF-α | 11.9 (6.2, 15.1) | 6.0 (2.9, 9.0) | **0.006** |
| TNF-α | 14.0 (11.8, 22.7) | 12.6 (10.0, 20.9) | 0.42 |
| TNF-β | 0.8 (0.8, 407.2) | 0.8 (0.8, 84.7) | 0.66 |
| VEGF | 458.6 (209.9, 798.5) | 268.9 (113.7, 408.8) | **0.043** |

^1^ Wilcoxon two sample test

Supplementary Table 12. Correlation of serum CCL22 levels with clinical features in patients at baseline

|  | Correlation with CCL22 level (Spearman’s *ρ*) | *p* value |
| --- | --- | --- |
| **BPRS Symptoms** |  |  |
| Delusions | 0.15 | 0.11 |
| Hallucinations | 0.20 | **0.032** |
| Disorganized speech | 0.23 | **0.010** |
| Blunted affect | 0.09 | 0.32 |
| **Functioning^1^** |  |  |
| SOFAS | -0.16 | 0.07 |
| **Cognition^1^** |  |  |
| Verbal performance | -0.23 | **0.021** |
| Visuomotor performance | -0.17 | 0.09 |

^1^ Correlations in controls: SOFAS *ρ* 0.05, *p*=0.60, verbal performance *ρ* -0.05, P=0.54, visuomotor performance *ρ* -0.09, *p*=0.30


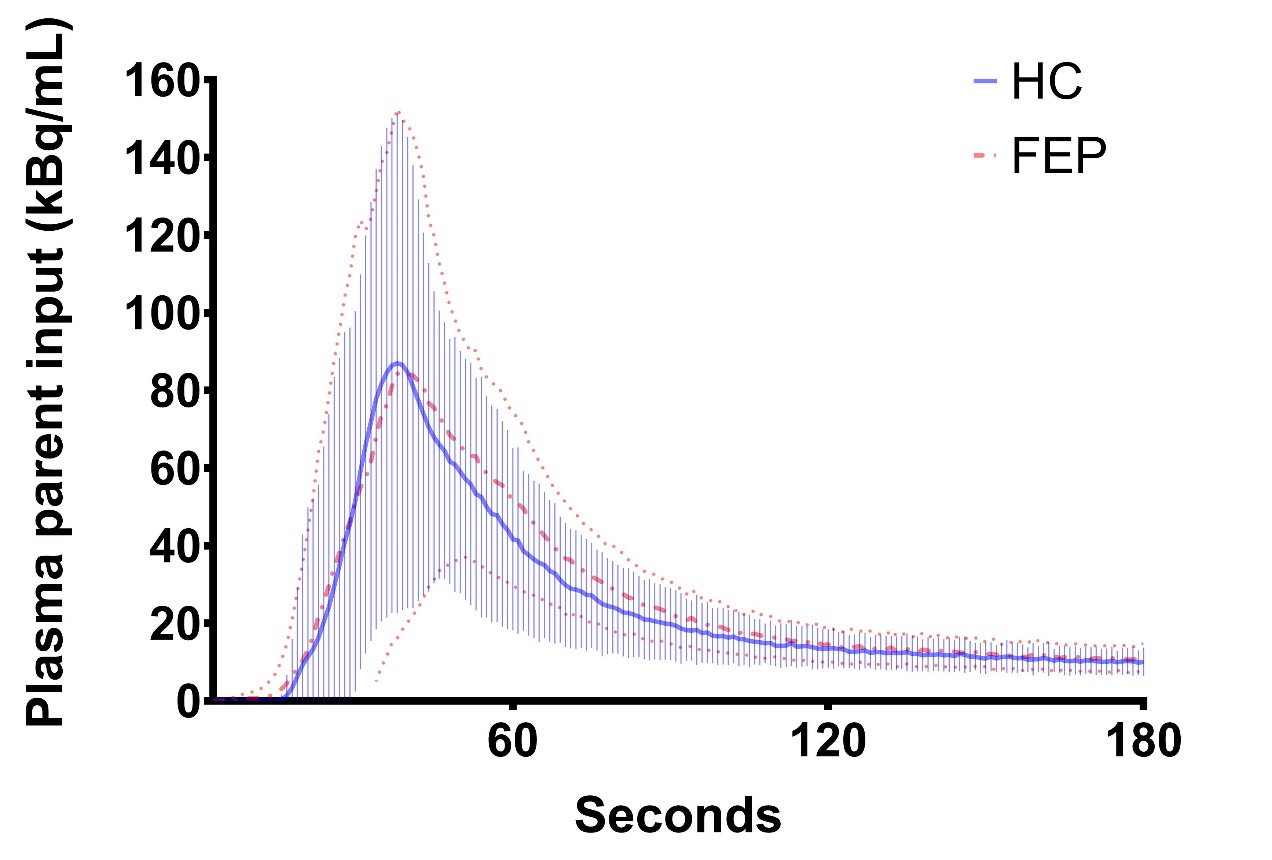
Supplementary Figure 1. Peak-phase time series of metabolite-corrected [^11^C]PBR28 plasma input in FEPs (n=13, red semi-dotted line) and HCs (n=15, blue solid line) separately. The red dotted line and blue bars indicate ±1 standard deviation around mean values in FEPs and HCs, respectively.


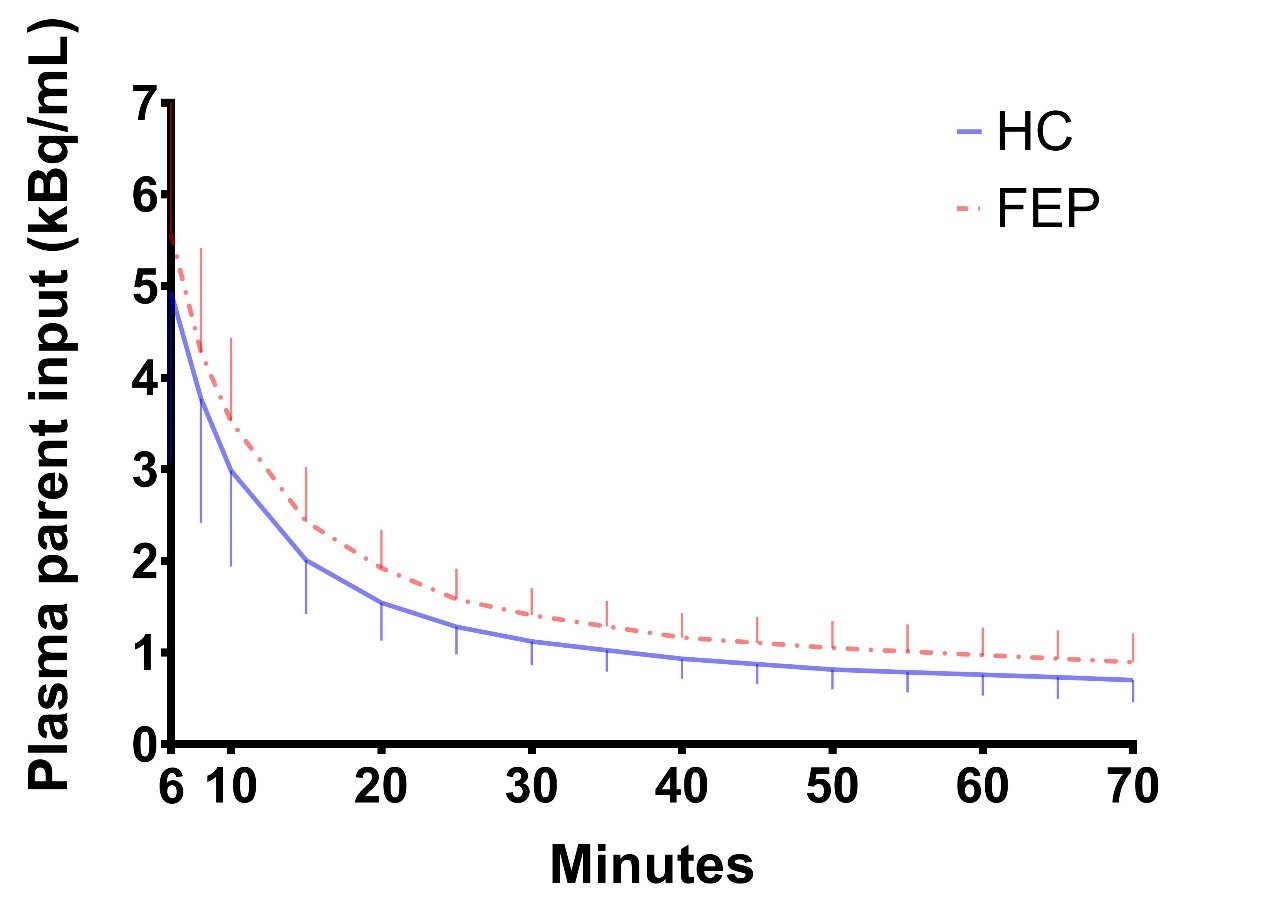
Supplementary Figure 2. Tail-phase time series of metabolite-corrected [^11^C]PBR28 plasma input in FEPs (n=13, red semi-dotted line) and HCs (n=15, blue solid line) separately. The red and blue bars ±1 standard deviation around mean values in FEPs and HCs respectively.


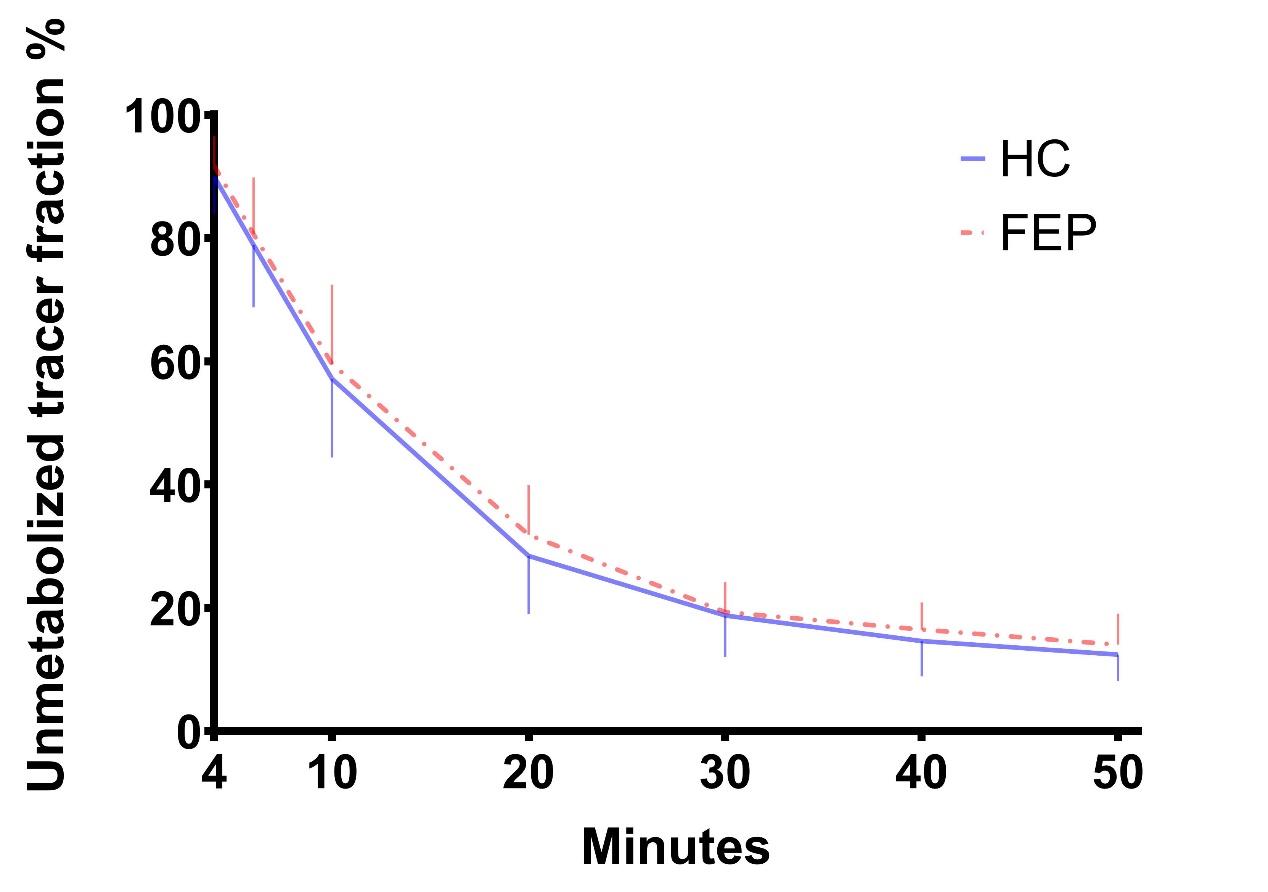
Supplementary Figure 3. Time series of unmetabolized [^11^C]PBR28 tracer fraction in FEPs (n=13, red semi-dotted line) and HCs (n=15, blue solid line) separately. The red and blue bars indicate ±1 standard deviation around mean values in FEPs and HCs respectively.


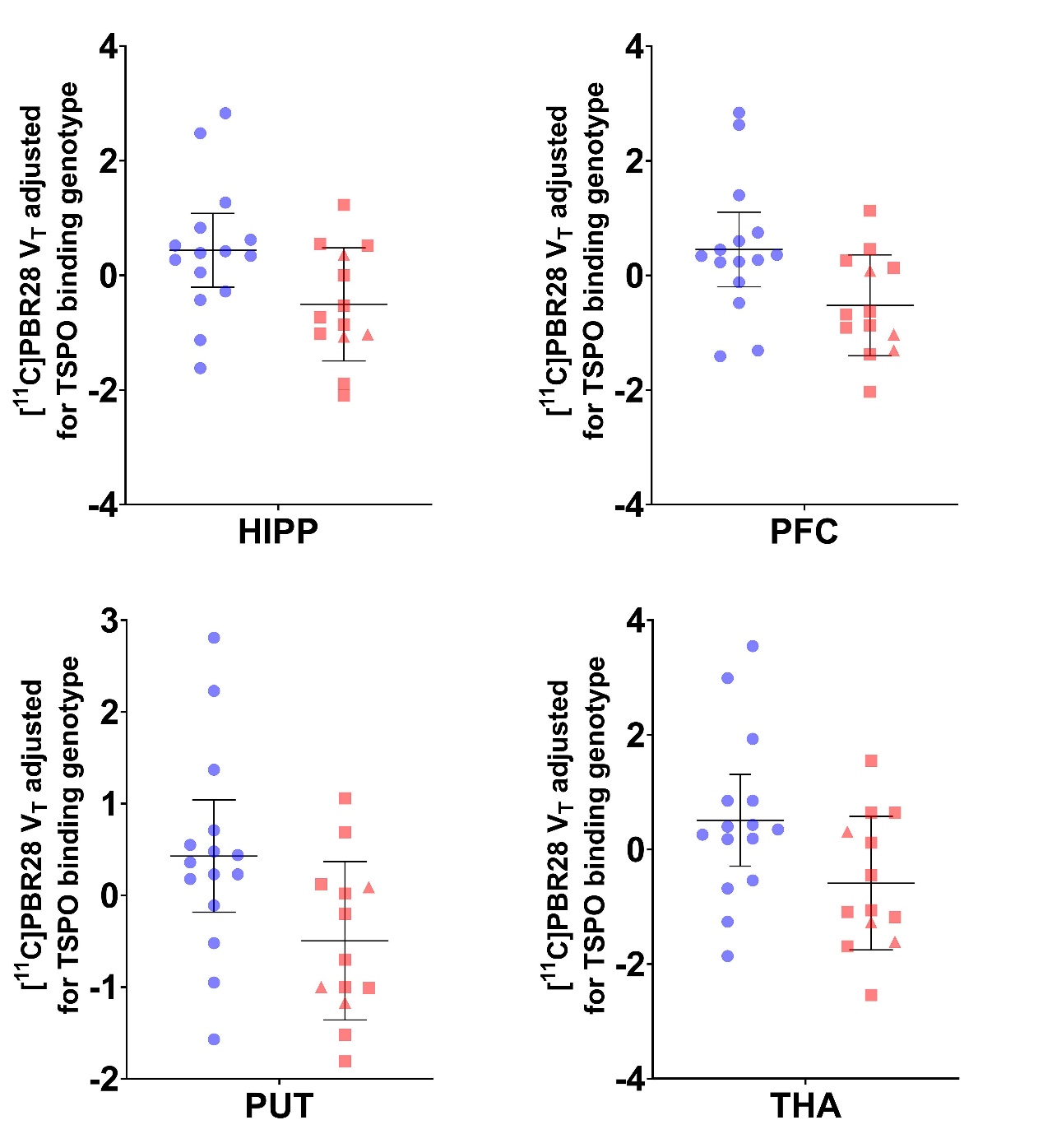


Supplementary Figure 4. Scatter plots show lower mean [^11^C]PBR28 V_T_ in FEPs (n=13) compared to HCs (n=15) in the hippocampus (HIPP, upper left), prefrontal cortex (PFC, upper right), putamen (PUT, lower left) and thalamus (THA, lower right). FEP subjects are denoted by red squares (non-affective psychosis) or red upwards triangle (affective psychosis), while HCs are represented by blue circles. Repeated measures analysis of variance with binding status as a covariate was used to compare regional [^11^C]PBR28 V_T_ between groups. Mean adjusted V_T_ is indicated with a long line in both groups separately. Error bars represent 95% confidence intervals.


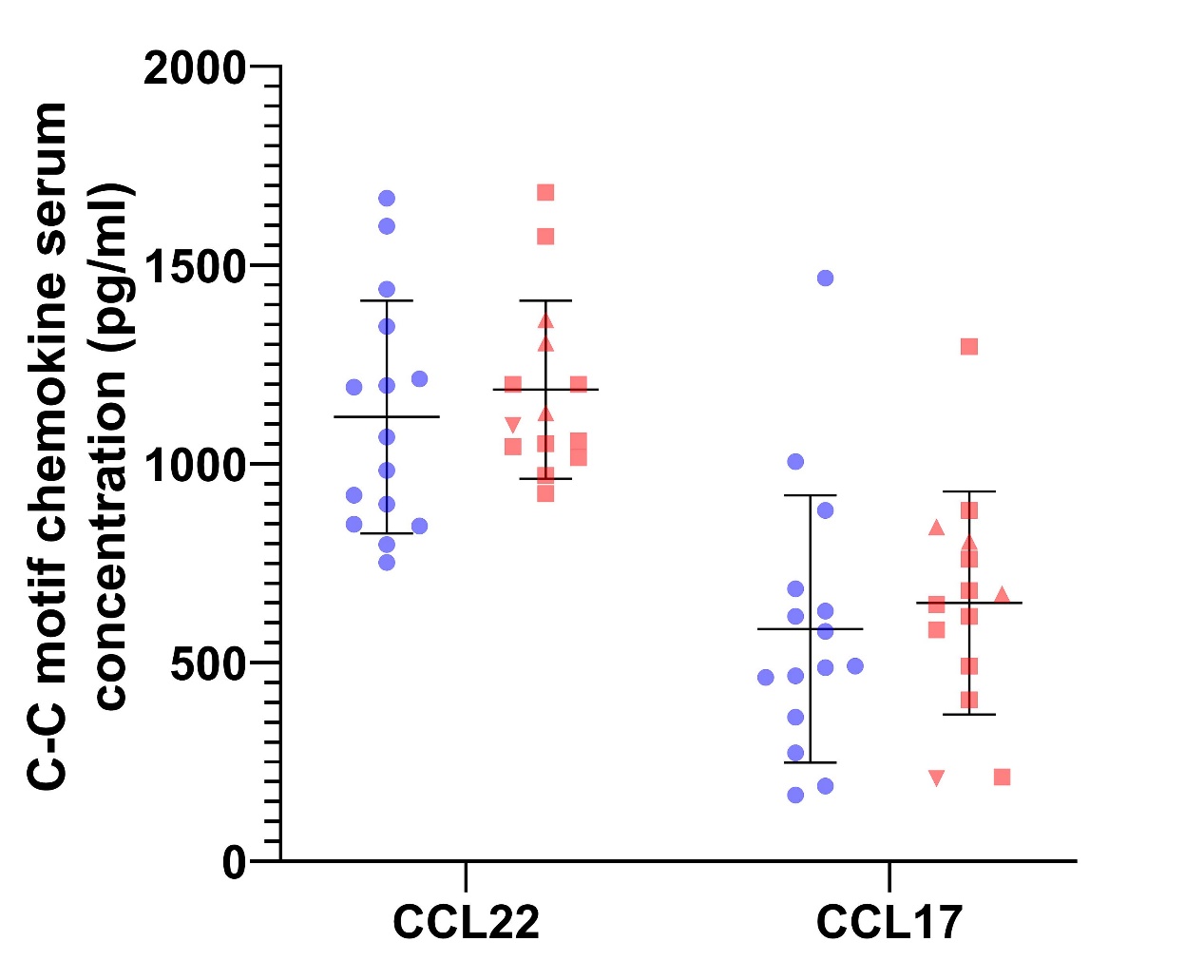
Supplementary Figure 5. Scatter plots show similar levels of CCL22 and CCL17 in first episode psychosis patients (FEP, n=14) compared to healthy controls (HC, n=15) included in the positron emission tomography study samples. Independent-samples t-tests were conducted to compare CCL22 and CCL17 concentrations between groups. FEP subjects are denoted by red squares (non-affective psychosis), red upwards triangles (affective psychosis), or a red downwards triangle (LAB TSPO genotype, affective psychosis). HCs are represented by blue circles. Mean concentrations are indicated with a long line in both groups separately. Error bars represent 95% confidence intervals.
